# Genome-wide identification, characterization, and expression profile of aquaporin gene family in flax (*Linum usitatissimum*)

**DOI:** 10.1038/srep46137

**Published:** 2017-04-27

**Authors:** S. M. Shivaraj, Rupesh K. Deshmukh, Rhitu Rai, Richard Bélanger, Pawan K. Agrawal, Prasanta K. Dash

**Affiliations:** 1 ICAR-NRC on Plant Biotechnology, PUSA, New Delhi, India; 2 Departement de Phytologie, University Laval, Quebec, QC, Canada; 3 National Agricultural Science Fund (NASF), PUSA, ICAR, New Delhi, India

## Abstract

Membrane intrinsic proteins (MIPs) form transmembrane channels and facilitate transport of myriad substrates across the cell membrane in many organisms. Majority of plant MIPs have water transporting ability and are commonly referred as aquaporins (AQPs). In the present study, we identified aquaporin coding genes in flax by genome-wide analysis, their structure, function and expression pattern by pan-genome exploration. Cross-genera phylogenetic analysis with known aquaporins from rice, arabidopsis, and poplar showed five subgroups of flax aquaporins representing 16 plasma membrane intrinsic proteins (PIPs), 17 tonoplast intrinsic proteins (TIPs), 13 NOD26-like intrinsic proteins (NIPs), 2 small basic intrinsic proteins (SIPs), and 3 uncharacterized intrinsic proteins (XIPs). Amongst aquaporins, PIPs contained hydrophilic aromatic arginine (ar/R) selective filter but TIP, NIP, SIP and XIP subfamilies mostly contained hydrophobic ar/R selective filter. Analysis of RNA-seq and microarray data revealed high expression of PIPs in multiple tissues, low expression of NIPs, and seed specific expression of TIP3 in flax. Exploration of aquaporin homologs in three closely related *Linum* species *bienne, grandiflorum* and *leonii* revealed presence of 49, 39 and 19 AQPs, respectively. The genome-wide identification of aquaporins, first in flax, provides insight to elucidate their physiological and developmental roles in flax.

Water absorption from soil through roots and its translocation to different parts is of paramount importance for innate physiological processes in plants. Within the plant system water movement takes place through apoplastic, symplastic and transcellular pathways^1^. Amongst the three defined pathways, transcellular movement of water in plants is facilitated by small integral membrane proteins (21–34 kD) called aquaporins (AQPs)^2^,^3^. These aquaporins belong to major intrinsic protein (MIP) superfamily with members spanning in animals^4^,^5^, plants as well as in microbes^6^. However, plants, unlike animals, harbor highly abundant and divergent AQPs^7^. Based on phylogenetic analysis, plant AQPs are grouped into five subfamilies: (i)plasma membrane intrinsic proteins (PIPs), (ii) tonoplast intrinsic proteins (TIPs), (iii) nodulin26-like intrinsic proteins (NIPs), (iv) small basic intrinsic proteins (SIPs), and (v) uncategorized X intrinsic proteins (XIPs)^8^,^9^,^10^,^11^. All AQP subfamilies are identified in different plant species including primitive land plant physcomitrella (*Physcomitrella patens*) except the XIPs that are absent in *Brassicaceae* and monocots^12^,^13^,^14^,^15^. In addition, two unique classes of AQPs, GlpF-like intrinsic protein (GIPs) and hybrid intrinsic proteins (HIPs) have been identified in physcomitrella^13^.

Primarily, plant AQPs are involved in water transport, though some of them are implicated in transport of small solutes such as urea, ammonia, glycerol, silicic acid, boric acid, CO_2,_ and H_2_O_2_^3^,^16^,^17^,^18^. AQPs from different plant species have also been reported to be involved in abiotic stress tolerance. Additionally, the role of AQPs in key developmental processes such as seed germination, stomatal movement, cell elongation, and reproductive growth including male fertility has also been established^19^,^20^,^21^,^22^,^23^. Understanding the multiple roles of AQPs have been facilitated by studying the interaction of AQPs with cognate solvents at molecular level. High resolution, three-dimensional structures of AQPs from different organisms including plants revealed their hourglass structure^24^. The structure of the protein is formed by six transmembrane (TM) α helices (helix 1 to helix 6), and five loops (loop A to E) that penetrates into the lipid bi-layer to make route for water transport. While, loops B and E contain highly conserved NPA (Asn-Pro-Ala) motifs in half-helices to form one of the two constrictions in the channel; the aromatic arginine (ar/R) region formed by each residue from helix 2 and helix 5, and two residues from loop E (LE1 and LE2) represent the other constriction. These two constrictions mostly determine the substrate permeability^24^,^25^,^26^,^27^. The NPA motifs are known to have role in plasma-membrane targeting for the AQPs and also shown to be involved in substrate specificity^28^,^29^. Recent study in plants highlighted the importance of the precise spacing between two NPA motifs in determining substrate specificity^30^. Change in amino acid residue at ar/R selectivity filters (SF), NPA motifs, and the spacing between NPA motifs are found to affect substrate specificity of AQPs in plants^28^,^30^,^31^. Similarly, few conserved amino acids known as Froger’s residues are found to be associated with substrate specificity particularly in microbial AQPs^32^. Experimentally validated structural and functional information of AQPs has led to identification of their orthologs in different plant species such as arabidopsis (*A. thaliana*)^10^, rice (*O. sativa*)^33^, poplar (*P. trichocarpa*)^34^, soybean (*G. max*)^35^,^36^ and tomato (*L. esculantum*)^37^.

Flax (*Linum usitatissimum*) is an important dual purpose industrial crop cultivated for high quality fibre (linen) and seed oil. The flax stem is the main source of cellulose rich bast fibre, used by textile industries for production of linen clothes. Its seed (linseed) oil is beneficial for human health owing to the presence of high amount of omega-3 fatty acids. Also, the oil of linseed is used in preparation of many industrial solvents. Considering the economic importance of flax, its genome was sequenced^38^ and subsequently there is an accumulation of genomic information in flax^39^,^40^,^41^. Water transporting aquaporin family members are implicated to play an important role in seed development and fiber formation in different crop plants^42^,^43^,^44^. In this study, genome sequence of flax was analyzed to identify the aquaporin encoding genes. This is the first report that identified 51 AQPs in flax through genome-wide analysis. Hitherto, flax aquaporins were classified based on the phylogenetic analysis with known AQPs, which showed five groups representing different sub-families of AQPs. Their distribution patterns, genetic architecture, structural properties and expression pattern were investigated to identify candidate AQPs with pivotal role in physiology and development in flax.

## Results

### Genome-wide distribution of Flax AQPs

Tabulation of blast output, BLASTp, employing AQP genes from ten different plant species as query (with high scoring pairs >100bit score) led to identification of 51 putative AQP genes (see Supplementary Table S1) in flax. While, domains of these putative proteins were analysed using conserved domain database (CDD) tool hosted in NCBI (see Supplementary Table S2), functional annotations of these sequences using protein database (PANTHER) confirmed function of these candidate sequences as aquaporins (see Supplementary Table S3). Protein domain analysis revealed the presence of six signature transmembrane domains (see Supplementary Table S4) in 35 out of the 51 identified AQPs. These AQPs were found to be distributed among 43 scaffolds (Table 1). Out of the 43 scaffolds, 35 contained only one AQP while eight scaffolds (scaffold 28, scaffold 59, scaffold 76, scaffold 123, scaffold 156, scaffold 280, scaffold 605 and scaffold 612) were found to harbor two AQPs each (Table 1).

### Phylogenetic distribution of AQPs in flax

**Figure 1:**
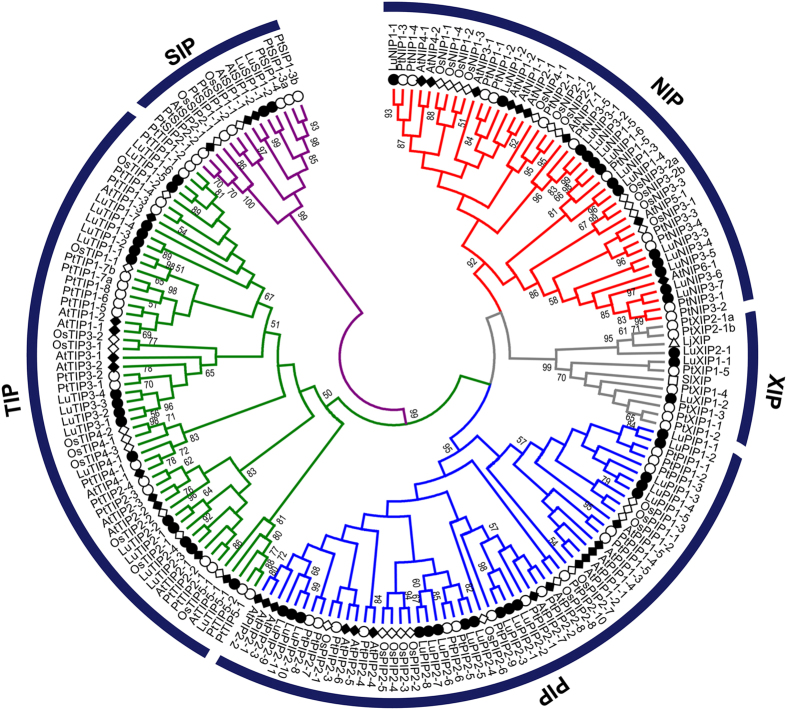
Phylogenetic analysis of flax aquaporins (AQPs) with rice. *Arabidopsis* and *Populus.* 16 PIPs, 17 TIPs, 13 NIPs, 2 SIPs and 3 XIPs were identified in flax genome and all the 51 AQPs grouped into five different classes such as PIPs, TIPs, NIPs, SIPs, and XIPs. The genes from rice, *Arabidopsis, Solanum,* *Lotus*, and *Populus* are indicated with the prefixes Os, At, Sl, Lj, and Pt respectively.

Phylogenetic tree of flax AQPs along with the known AQPs from *A. thaliana, O. sativa* and *P. trichocarpa* showed five distinct clusters representing different class of AQPs (Fig. 1). The flax AQPs were named according to their grouping with known AQPs and were classified into 16 LuPIPs, 17 LuTIPs, 13 LuNIPs, 2 LuSIPs and 3 LuXIPs. Within the groups formed by flax AQPs, two major subgroups were found in LuPIPs (LuPIP1, LuPIP2). Among them, LuPIP1 represented five members while LuPIP2 comprised of 11 members. Similarly, the LuNIPs formed two subgroups of LuNIP1 and LuNIP3 comprising of six and seven members respectively. Surprisingly, the NIP2 subfamily was found to be missing in flax genome. LuTIPs formed five subgroups (Fig.1) with LuTIP1 having seven members, LuTIP2 and LuTIP3 having four members each and, LuTIP4 and LuTIP5 having one member each. LuXIPs formed two subgroups, LuXIP1 comprised of two members and LuXIP2 with one member. LuSIPs did not form any subgroup except two isoforms SIP1-1 and SIP1-2. In our study, although homologs of all TIP, PIP and XIP family members have been identified, the homologs of NIP2 and SIP2 were not found.

### Silicon accumulation in flax plants

**Figure 2:**
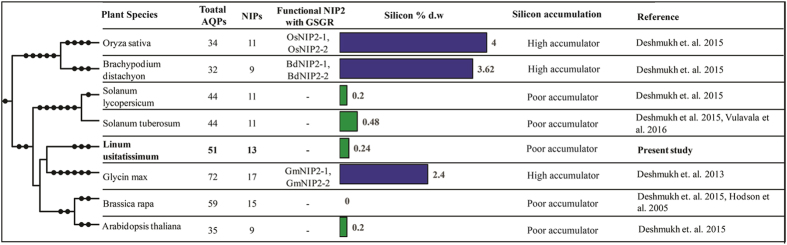
Estimation of silicon content in flax. Si content in flax was compared with Si accumulation in other reported plant species such as rice (4%) and Brachypodium (3.6%) and are classified as high Si accumulators and possess NIP2 while crops like tomato, potato (0.2–0.5%) and flax (0.24%) are classified as poor accumulators and are devoid of NIP2s.

The NIP2s having signature sequence of Gly-Ser-Gly-Arg (GSGR) ar/R selectivity filter are well characterized as a silicon transporters. On the basis of presence or absence of NIP2s a previous study^30^ had characterized 25 plant species as poor or rich silicon accumulators. Results of phylogenetic analysis and subsequent identification of conserved motifs and ar/R selectivity filter revealed loss of Si transporting signature (GSGR) containing NIP2 members in flax. We validated the effect of loss of NIP2s in flax by measuring the silicon accumulation capability in flax shoot (Fig. 2). Quantification of silicon in one-month old flax plant revealed 0.24% silicon on dry weight basis in the leaf tissues. Low accumulation of Si in flax corroborated absence of NIP2 in flax. Further, comparison with previously reported data grouped flax into poor silicon accumulator category along with *Arabidopsis thaliana*^30^, *Brassica rapa*^45^ and *Solanum tuberosum*^46^ all of which have less than 1% silicon accumulation when grown with continuous supply of sufficient silicon (1.7 mM). All these poor silicon accumulator plants are devoid of NIP2s whereas the high silicon accumulator plants like soybean^35^, *Brachypodium*^30^, and rice^30^ possess NIP2s and are found to be accumulating over 2.4% silicon on dry weight basis (Fig. 2).

### Gene structure, organization and evolution of flax AQPs

**Figure 3:**
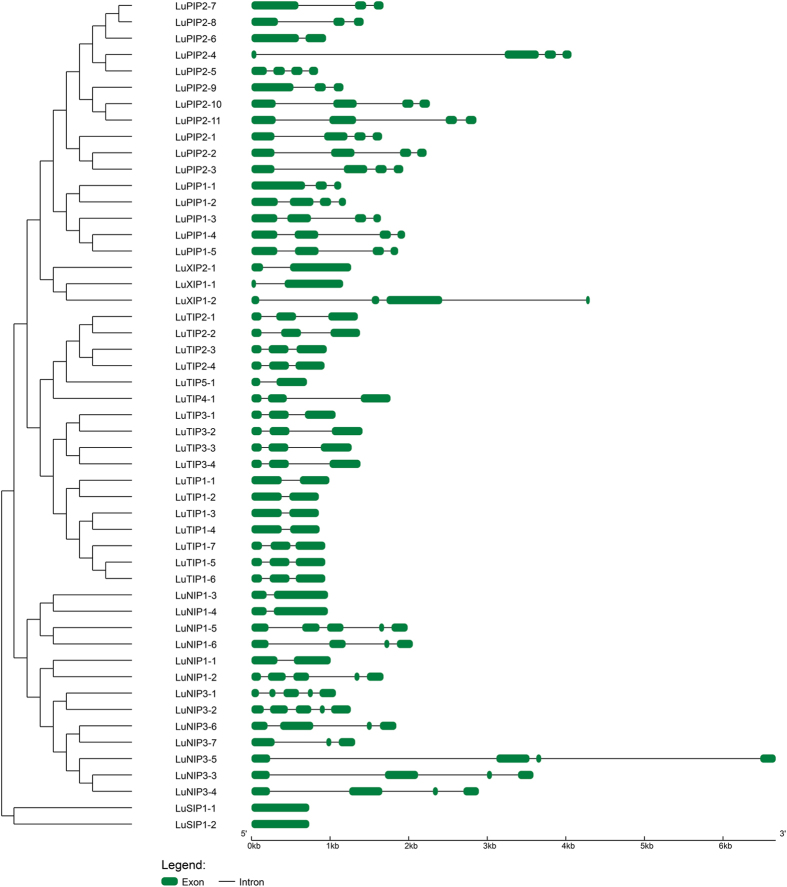
Analysis of exon-intron structure of flax aquaporins (AQPs). Graphic representation of the gene models of 51 AQPs identified from flax genome revealed presence of varied number of introns (0–4). Exons are shown as green boxes and introns are shown as black lines. Length of the exon and intron (bp) is indicated in kb in x-axis.

Flax AQPs showed less variation in transcript length (ranging from 498 bp to 945 bp) compared to variation in gene length (ranging from 706 bp to 6670 bp). Exon intron structure analysis depicted presence of varied number of introns among the AQPs contributing to the variation in gene length (Fig. 3). Our study revealed that the number of introns in flax AQPs ranged from zero (LuSIP1-1; LuSIP1-2) to four (LuNIP1-2, LuNIP1-5, LuNIP3-1, LuNIP3-2) introns. While, both SIP homologs were found to be intronless; least number of introns were observed in TIPs (1-2) followed by PIPs (1-3) and NIPs (1-4). The exon-intron features observed in flax AQPs were similar to gene structure of AQPs observed in other crop plants. Of the 17 TIPs, maximum number of homologs (12) contained two introns while five homologs harbored single intron each. Similarly, of the sixteen PIPs identified in flax genome ten homologs contained three introns, while rest six PIPs contained less than three introns. Among the NIPs, maximum numbers of NIPs (9) harbored either three or four introns. Conserved intron-exon organization of AQPs in the four subfamilies suggest diversification of AQPs occurred early before the evolution of flax. The identified AQPs from flax showed amino acids ranging from 165 (LuTIP5-1) to 314 (LuXIP1-2) and the molecular weight of proteins ranged from 16.80 kD (LuTIP5-1) to 32.65 kD (LuPIP1-1).

The distinct pattern of intron-exon organization structure observed amongst flax AQPs correlated well with their phylogenetic distribution (Fig. 3). Most of the phylogenetically related AQPs shared similar gene organization suggesting possible gene duplication event. Unlike previous report of presence of 2-3 introns in SIPs, both the SIP genes in flax were devoid of introns. Similarly, two groups of SIPs are reported in soybean, rice, arabidopsis and chickpea^30^,^47^ while SIP2 is missing in flax. It suggests, SIPs are evolutionary more vulnerable in flax. Additionally, distribution of selectivity filters also resembled well with the phylogenetic distribution in flax AQPs. The NIP groups posses relatively less conserved selectivity filter compared to groups in the other AQP subfamilies suggesting less selection pressure on NIPs.

### Characterization of NPA motif, transmembrane domains and sub-cellular localization of flax AQPs

The Flax AQPs displayed difference in Asn-Pro-Ala (NPA) motifs and residues at ar/R selectivity filters and Froger’s positions (Table 2). Most of the AQPs contained dual NPA motifs except LuPIP2-4, LuPIP2-5, LuTIP5-1, and LuNIP1-6 which were found to harbor single NPA motif. Majority of the members from PIP and TIP sub-family showed typical NPA motif as observed in *A. thaliana* counter part except LuPIP2-9 and LuTIP1-6 which showed Asparagine to Glycine and Asparagine to Proline substitution respectively, in the first NPA motif. The GPA (LuPIP2-9) and PPA (LuTIP1-6) motifs observed in flax were not reported in any other plant species. Such changes are expected to alter the substrate specificity of the aquaporins in flax. In the NIP sub-family, the first NPA domain was found to be conserved in all the members, while second NPA motif showed Alanine to Valine substitution in five members of LuNIP sub-family (LuNIP3-3 to LuNIP3-7). In SIP and XIP sub-family first NPA motif showed substitution, while second NPA motif was found conserved. Similarly, first NPA motif of SIP sub-family harbored threonine in place of alanine; while isoluecine (LuXIP1-2) or valine (LuXIP1-1, LuXIP2-1) substituted alanine in XIP sub-family in the same motif.

**Figure 4:**
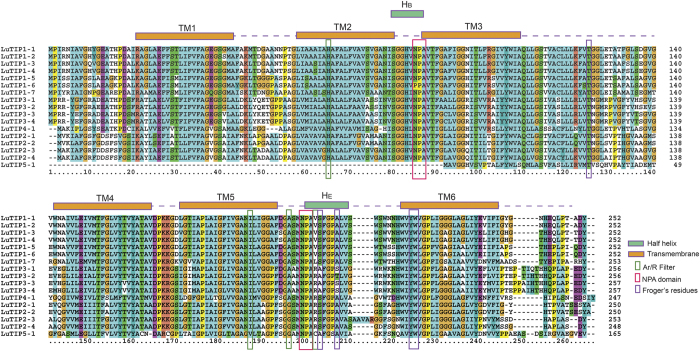
Protein sequence alignment of TIPs. Conserved transmembrane domains (TM1-5) and amino acids at NPA domains, ar/R selectivity filters, and Froger’s residues identified in five TIP family members (TIP1 to TIP5) in flax.

All the PIP sub-family members showed a conserved ar/R filter residues with Phenylalanine in H2, Histidine at H5, Threonine at LE1 and Arginine at LE2 (see Supplementary Fig. S1). In TIP sub-family H2 position of ar/R filter comprised of Histidine, H5 position comprised of Isoleucine except for LuTIP5-1, which contains Valine residue (Fig. 4). LE1 and LE2 positions were found to be specific for each subgroup of LuTIPs. LuTIP1 subgroup was mostly characterized by Alanine (LE1) and Valine (LE2) except LuTIP1-7 that contained Leucine (LE2). LuTIP2 subgroup is characterized by Glycine (LE1) and Arginine (LE2). LuTIP3 and LuTIP4 sub-groups were characterized by Alanine (LE1) and Arginine (LE2). In NIP sub-family, the NIP1s were characterized by Tryptophan (H2), Valine (H5), Alanine (LE1) and Arginine (LE5) whereas the NIP3s were comprised of Alanine/Serine/Threonine (H2), Valine/Isoleucine (H5) Glycine/Alanine (LE1) and Arginine (LE2). The SIP family members showed Alanine/Isoleucine/Proline/Asparagine residues whereas XIP sub-family members showed Isoleucine/Valine (H2), Luecine/Valine (H5), Valine/ Arginine (LE1), Arginine (LE2) (see Supplementary Fig. S2).

To ascertain expression of flax AQPs at different cellular/ organellar levels, their sub-cellular localizations were predicted (Supplementary Table S5). Majority of flax PIPs were predicted to localize in the plasma membrane. Out of seventeen TIP subfamily members, only two were predicted to localize in plasma membrane, while nine TIPs were targeted to cytoplasm and five were targeted to vacuoles. Majority of NIPs were predicted to be associated with plasma membrane. While SIPs localized into the vacuoles, XIPs localized into the plasma membrane or cytoplasm.

### Identification of Aquaporins in different *Linum* species

RNA-seq data for three different *Linum* species such as *L. bienne, L. grandiflorum* and *L. leonii* were downloaded from short read archive (SRA) at NCBI and analysed with an aim to identify orthologs of aquaporins (see Supplementary Fig. S3, Table 3). The *de novo* assembly of RNA reads showed N50 values of 520 bp, 797 bp and 1254 bp for *L. bienne, L. grandiflorum* and *L. leonii*, respectively (see Supplementary Table S6). In comparison to *L. usitatissimum*, forty-nine aquaporins were observed in *L. bienne* while thirty-nine aquaporins were found in *L. grandiflorum* and nineteen AQPs were observed in *L. leonii*. Amongst forty-nine aquaporins found in *L. bienne,* twenty-nine were PIPs, twelve were TIPs, five were NIPs, two were SIPs, and one was XIP. Expansion of PIP specific family members was observed in *L. bienne* with twenty-nine PIPs as compared to sixteen PIPs in *L. usitatissimum*. Comparable number of PIP family members with fifteen and eleven PIPs was also observed in *L. grandiflorum*, and *L. leonii* respectively. The phylogenetic analysis of AQPs identified from different *Linum* species showed grouping in accordance to their sequence homology (see Supplementary Fig. S3B).

### AQP expression profiling in flax

In order to provide transcriptional evidence, homology search was performed against flax specific ESTs at the NCBI database (http://blast.ncbi.nlm.nih.gov/) that revealed existence of ESTs for 31 out of the 51 identified AQPs (Table 1). The highest numbers of ESTs were found for LuTIP3-3 (91), LuTIP3-4 (80) and LuPIP2-1 (52), whereas, least number of ESTs (0-1) were observed for the NIP family members among the different groups of AQPs.

**Figure 5:**
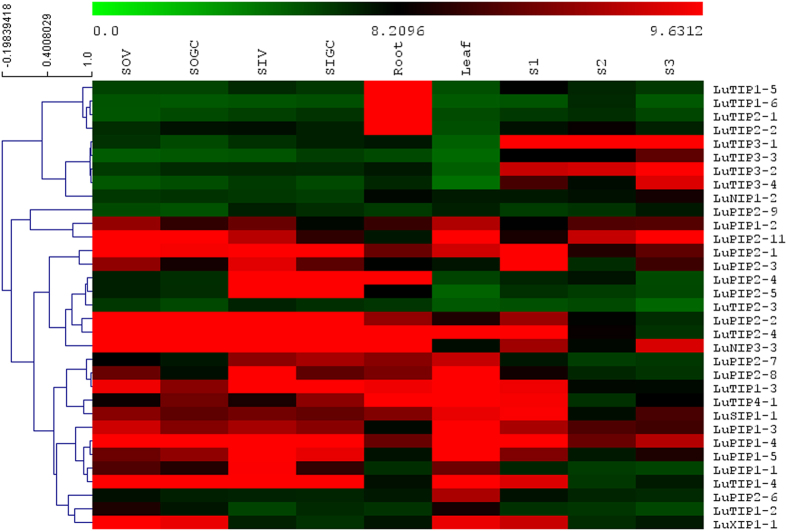
Analysis of flax aquaporins expression using microarray data^48^. Majority of PIPs showed higher level of expression compared to other MIPs across multiple tissues in flax.The different tissues included for expression analysis are root, leaf, stem inner at vegetative stage (SIV), stem inner at green capsule stage (SIGC), stem outer at vegetative stage (SOV), stem outer at green capsule stage (SOGC), developing seed 10 DAF (S1), 20 DAF (S2), 40 DAF (S3).

Analysis of *a priori* reported microarray data^48^ revealed the expression of thirty three AQPs out of 51 AQPs represented on the array (Fig. 5). Among different AQPs, majority of the TIPs showed low expression whereas many PIP family members showed higher expression across nine different tissues of flax such as root, leaf, stem (stem inner at vegetative stage, stem inner at green capsule stage, stem outer at vegetative stage, stem outer at green capsule stage) and developing seed at 10 days after flowering (DAF), 20DAF and 40DAF. The expression was calculated in terms of fold change of AQPs in different tissues of flax in comparison to root revealed similar results (see Supplementary Fig. S4). LuTIP3-1, LuTIP3-2, LuTIP3-3 and LuTIP3-4 showed higher accumulation in seeds as compared to other tissues. Majority of PIPs showed higher expression in leaf, root, stem, and initial stage of embryo development (10 DAF) while low expression was observed during later stages of embryo development (20 DAF and 40 DAF). While, PIP1s (PIP1-3 to PIP1-5) and PIP2s (PIP2-1, PIP2-2, PIP2-4) showed higher expression in both outer and inner stem, few PIP2s (PIP2-5, PIP2-11) showed differential expression in outer as well as in inner stem during vegetative and green capsule stage in flax.

**Figure 6:**
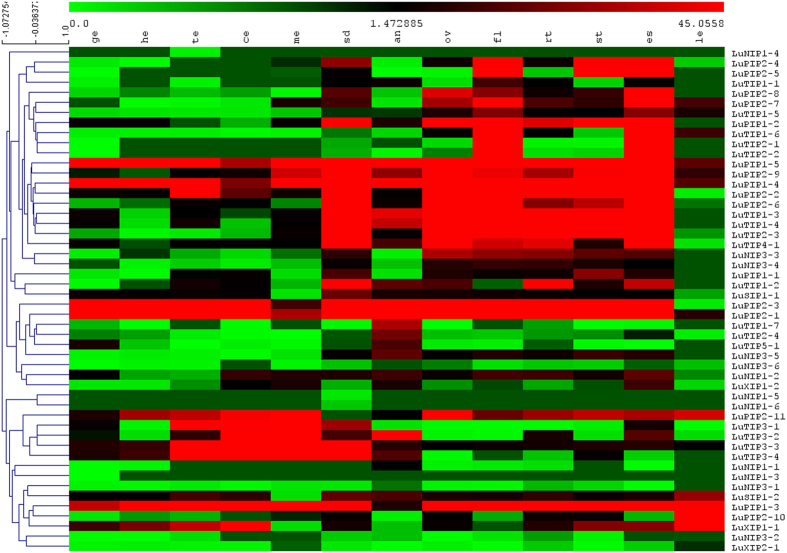
Analysis of flax aquaporins expression using RNA-Seq data^77^. PIPs show higher accumulation and NIPs show low expression across multiple tissues in flax. The different tissues included for expression analysis are globular embryo (ge), heart embryo (he), torpedo embryo (te), cotyledon embryo (ce), mature embryo (me), seeds (sd), anthers (an), ovaries (ov), mature flower (fl), root (rt), stem (st), etiolated seedlings (es), leaves (le).

**Figure 7:**
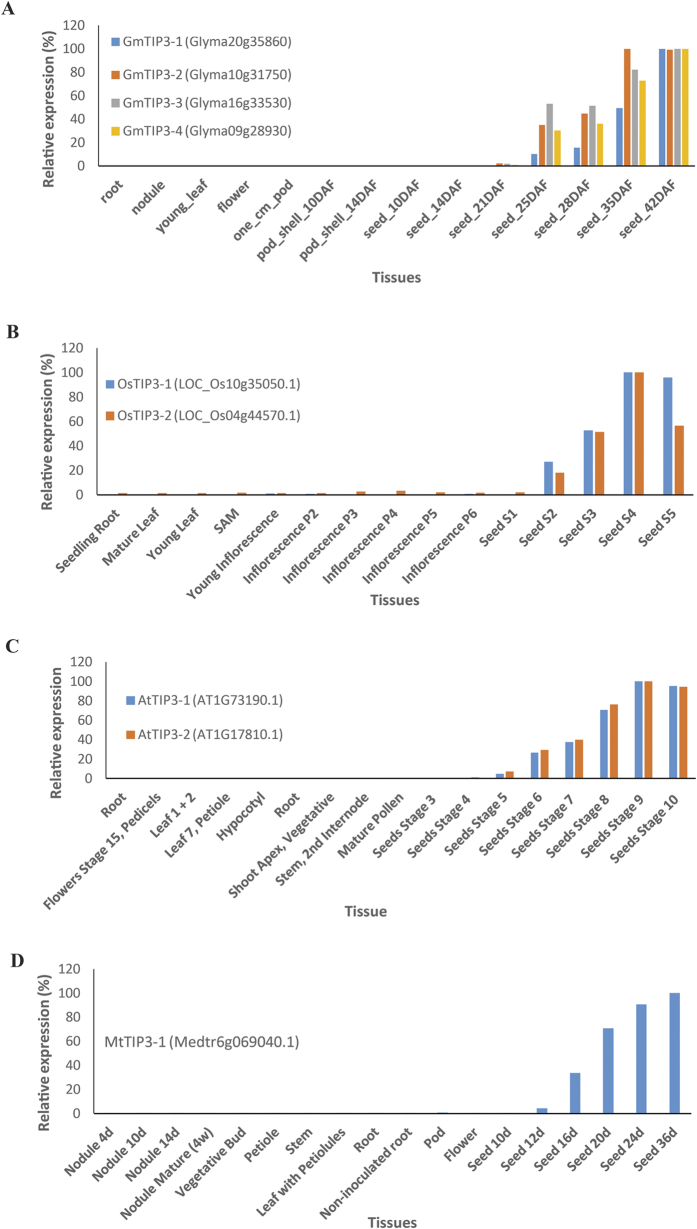
Expression profiling of TIP3 genes across different tissues and developmental stages in multiple crops. (**A**) Soybean, (**B**) Rice, (**C**) *Arabidopsis*, and (**D**) *Medicago*. Higher levels of TIP3 transcripts are found in seeds compared to different tissues and developmental stages across all the four plant species analyzed.

The RNA-seq data was congruent with expression signatures observed in microarray data-set. The majority of PIPs showed higher level of expression across thirteen different tissues (Fig. 6). All NIP homologs were well represented in the RNA–seq dataset and majority of them showed lower accumulation in different tissues compared to other family members. Among TIPs, TIP3 members (LuTIP3-1, LuTIP3-2 and LuTIP3-3) showed gradual increase in expression from globular stage of embryo to mature embryo stage during seed development in flax. The expression was also calculated in terms of fold change of AQPs in different tissues in comparison to root (see Supplementary Fig. S4). Similarly, in other plant species such as soybean, rice, *Arabidopsis* and *Medicago*, expression of TIP3s specific to seed tissue was observed (Fig. 7). The pattern of TIP3s expression gradually increasing from early stage of seed development to maturation was commonly observed in all species analyzed (Fig. 7).

Differential expression of genes at apical and basal region of flax stem was delineated from another set of RNA-seq transcriptome profiling^49^. Comparison of expression pattern of AQPs in apical and basal region of flax stem identified eighteen AQPs with two fold transcript enrichment in apical region (AR) compared to basal region (BR) (see Supplementary Table S7). The differentially expressing 18 AQPs comprised of seven PIPs, five TIPs, five NIPs and one XIP. Among different sub-family of MIPs, members of PIP subfamily, PIP2-4, PIP2-5 and PIP2-7 showed higher expression levels with 12, 11 and 5.3 fold respectively in AR compared to BR.

### Analysis of tertiary protein structure of flax AQPs

**Figure 8:**
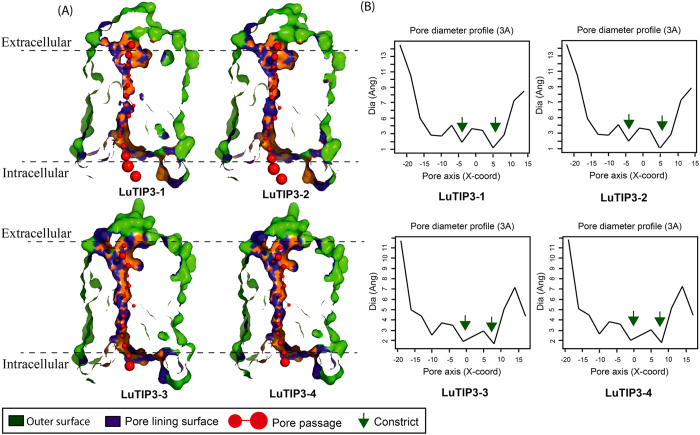
Pore morphology and dimensions of flax TIP3s. Protein tertiary structure showing pore morphology of LuTIP3 (**A**) family members. Cross section of the proteins showing pore is depicted for each family member along with the graph showing pore dimensions obtained from PoreWalker software (**B**).

Homology based tertiary (3D) protein structure of all 51 flax AQPs predicted to form hourglass like structure with six transmembrane domains (see Supplementary Fig. S5). Pore structure and three dimensional geometry of a channel of TIP3 (TIP3–1, TIP3–2, TIP3–3, TIP3–4) family members obtained with “PoreWalker” software identified a pore that longitudinally runs from the extracellular to intracellular opening of the protein. The pore morphology clearly showed conservation of pore size and two constrains that are known to act as selectivity barrier in the pore (Fig. 8). Though, PoreWalker analysis does not provide information about solute interaction, the data of pore morphology obtained with it helps to predict the solute permeability. Conservation of pore size and similar constrain in all the four TIP3s indicates its plausible role in water transport. Similarly, pore structure and three dimensional geometry of PIP2s (PIP2-4, PIP2-5 and PIP2-7) family members of flax obtained with “PoreWalker” software showed conservation for pore size and constrains in the pore (see Supplementary Fig. S6).

## Discussion

Aquaporins are the key membrane transport proteins involved in transport of water and substrate in the plant system. Plants have, relatively, a high number of aquaporins that are evolved into specific subfamilies and groups comprising constitutively expressed, tissue-specific, temporal or environmental factors and stress induced AQPs. Recent studies also revealed role of AQPs in abiotic stress tolerance in arabidopsis and barley plants^3^,^50^,^51^. Recent spurt of decoding whole genome sequence of crops has led to the identification of varied gene families including AQPs in many plant species. The available draft genome sequence of flax provided us an opportunity to analyze the AQP gene family in flax *vis-a-vis* other taxa including rice, poplar and *Arabidopsis*. We identified 51 putative AQPs in flax genome, which is more than the number of AQPs identified in rice (34) and *Arabidopsis* (35). Additionally, AQPs search performed using RNA-seq data in the three *Linum* species identified similar number of aquaporins in *L. bienne* having comparable chromosome number (n = 15) as flax. The *L. grandiflorum* (n = 8) and *L. leonii* (n = 9) showed less number of aquaporins besides having much longer contigs than the *L. bienne* with *de novo* assembly. The variation in the AQPs across the *Linum* species could be due to the differences in the genome size. However, there is a possibility of identification of more number of AQPs in these species with the sequencing of more whole genomes. In particular, the NIPs with low tissue specific expression can be a serendipitous discovery. The number of AQPs observed in flax was found to be similar to poplar (55) which is also a member of the order malpighiales. Whole-genome duplications and inter-specific hybridizations have resulted in expansion of gene copy number in plants. Thus, the presence of more number of AQPs in flax compared to *Arabidopsis* and rice is attributed to recent whole-genome duplication event that occurred about 5–9 MYA in flax lineage, after it’s divergence from poplar and castor^38^. Since, chromosome-scale assembly is not available in flax, the assembled sequence in the form of scaffolds was helpful to locate tandem duplications. However, analysis of genomic distribution of AQPs revealed the absence of tandem duplications among the flax AQPs.

As observed in most of the plant genomes, flax AQPs grouped into five sub-families (PIPs, TIPs, NIPs, SIPs, XIPs) except monocots and *Brassicaceae* which harbor four AQP (PIPs, TIPs, NIPs, SIPs) sub-families^13^,^14^. Number of flax AQPs in different sub-families was also similar to that of *Populus*. However, the number of XIPs and SIPs varied in both genomes compared to other sub-families. Six members each of SIPs and XIPs were reported in *Populus*, while in flax two SIPs and three XIPs were observed. Specifically, members of SIP2s and NIP2s were not observed in flax genome while NIP2s were also absent in *Arabidopsis* genome. The exon-intron distribution in members of flax AQP subfamilies were found to be similar to the gene structure of AQPs observed in other crop plants^35^,^47^. Similar gene structure indicates conserved function of AQPs in flax. It is well documented that the intron number is correlated with the gene expression, gene duplication, and diversification^52^ of genes in plants.

Usually, the substrate specificity of the AQPs is determined by the hydrophobicity and size of the amino acids forming the pores^24^,^26^,^27^. These positions in flax AQPs are based on the previously known AQPs from other plant species. Two highly conserved NPA motifs in loops B and E along with four amino acid residues forming aromatic/arginine filter determine the transport of substrate molecule. All PIP family members from flax contained more hydrophilic ar/R selectivity filter (F/H/T/R) a hallmark of water transporting aquaporins compared to other families. Similar ar/R selectivity filter was also observed in PIP family of aquaporins from other plant species such as *A. thaliana, B. rapa, G. max, P. vulgaris*, and *R. communis*^10^,^35^,^36^,^53^,^54^,^55^. The water transporting AQP1 from humans also contains a similar ar/R selectivity filter with large hydrophilic amino acid residues (F/H/C/R). PIPs are known to play a central role in transport of water regulating root and leaf hydraulics^3^. In addition to water transport, PIPs are known to facilitate diffusion of CO_2_ in mesophyll tissue of *A. thaliana* and *N. tabacum* affecting photosynthesis^56^,^57^. Our expression analysis also showed abundant expression of PIPs in flax root, stem as well as leaves suggesting possible role of PIPs in water transport and CO_2_ diffusion in flax.

Among LuTIPs, LuTIP1s were found to have residues (H/I/A/V, H/I/A/L) forming more hydrophobic ar/R filter compared to LuTIP2s and LuTIP3s which contained ar/R filter with H/I/G/R and H/I/A/R residues respectively. The residues present in ar/R selectivity filter in LuTIP subfamily were similar to TIPs from other plant species. TIPs are located mainly in vacuolar membrane and act as functional water transporters. A number of experiments have shown the ability of TIPs to facilitate transport of small solutes such as 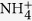, H_2_O_2_, and urea^58^,^59^,^60^. Conserved structural motifs such as NPA motif and ar/R filter in TIPs involved in transport of water as well as substrate were also observed in flax.

Among the NIPs, NIP1s were found to be more hydrophobic (WVAR) compared to NIP3s (AVGR, SIAR, TIAR). Interestingly, in the present investigation, members of NIP2 sub-group were not observed in flax genome. In plant kingdom different species accumulate wide range of silicon^61^. The ability of plants to absorb Si is attributed to the presence of NIP2s containing GSGR selectivity filter with a precise distance of 108 amino acids distance between the NPA domains^30^,^62^,^63^. Plant species considered as high accumulators of Si are known to accumulate up to 10% of Si on dry weight basis^64^. The low accumulator plants lacking NIP2s or functional NIPs without precise distance between NPA domains accumulate around 0.2% or less of silicon. Thus, less accumulation of Si in flax leaves is possibly due to absence of NIP2 members. NIPs exhibit low level of expression compared to PIPs and TIPs and their expression is confined to specialized cells and organs^65^,^66^. Low expression of NIPs as observed in microarray and RNA-seq data of flax was also supported by least number of ESTs found in the flax EST database.

The variations in ar/R selective filter for XIP family members specifically at H5 position have been reported in different studies^13^,^34^. In one study, Serine/Threonine residue was reported at H5 position in plants^34^; while in other study Valine/Isoleucine was reported^13^. The ar/R selectivity filter in XIPs from different plants is more hydrophobic in nature, while Valine/Isoleucine at H5 position increases its hydrophobicity. In flax,Valine/Leucine imparting more hydrophobicity occupies H5 position. This hydrophobic nature of XIPs facilitates transport of bulky and hydrophobic molecules such as glycerol, urea, and boric acid in plants^67^.

Analysis of microarray and RNA-seq data *in toto* revealed, both were congruent, higher accumulation of TIP3 specific transcripts in developing seeds of flax. TIP3s are generally involved in cellular osmoregulation and maturation of the vacuolar apparatus to support optimal water uptake and growth of the embryo during seed development and germination. Increasing level of TIP3 expression from early stages to seed maturation also suggests its role in seed desiccation process. Similar observations have been noted during seed maturation and initial phase of seed germination in *Arabidopsis*^39^ and seed specific expression in soybean^35^,^42^. Differential expression analysis^49^ between apical and basal region of flax also identified many aquaporin genes in fiber development. Contrary to role of TIP3 in seed development, PIP2s are envisaged to control fibre length in cotton^43^ by mediation of turgor pressure in developing fibres. Among aquaporin encoding genes, PIP2s showed higher differential expression in flax.

Recently, AQPs are envisaged to control fibre length in cotton^43^. Rapid elongation of cotton fibres is accomplished by the coordinated action of turgor potential across the tonoplast that pushes against and loosens the cell wall of fibre initials. During fibre elongation, enhanced turgor potential is generated by accumulation of sugar, malate and K^+^ besides influx of water by AQPs^68^,^69^. Panoply of genes involved in osmoregulation and cell expansion during fibre elongation in cotton has been identified. Phylogenetically, AQPs of cotton involved in fibre development belong to five subfamilies (PIP, TIP, NIP, SIP, and unrecognized intrinsic proteins XIP^70^) of which PIP2s are up-regulated during fibre development. PIP2s inflict rapid influx of water by resulting high turgor pressure that accentuates longitudinal and polar expansion of cotton fibre primordia. Precise role of PIP2s was further proved by developing RNAi transgenic plants targeting PIP2 that exhibited “Short-fibre phenotype” with >20% reduction in fibre production in cotton^43^. It is reported that in the short fiber mutant of cotton “Ligon lintless”^71^,^72^,^73^ most of the AQPs such as PIP (seven genes), TIP (four genes) and NIP (two genes) are massively down-regulated (p < 0.0001) compared to the wild type *G. hirsutum*. Nonetheless, equivalent information is meager to ascertain the role of AQPs in bast fibre development in flax. Thus, the role of aquaporins in flax fiber development needs further investigation.

## Conclusions

To the best of our knowledge, this is the first comprehensive genome-wide analysis of the AQP gene family in flax. The sequence comparison, phylogenetic analysis and expression analysis of AQPs in flax revealed presence of flax AQPs clustering into five sub-families. The global expression profiles of 51 AQP genes through microarray and RNA-seq data analysis revealed TIPs exhibit lower expression while PIPs exhibited higher expression in flax. The RNA-seq data precisely pointed out low expression of NIPs in multiple tissues compared to other AQPs. A gradual increase in TIP3 expression was observed from globular stage till seed development in flax envisaging a pivotal physiological role of TIP in seed development. Further, absence of NIP2 AQP in flax was observed and was commensurate with low accumulation of silicon in flax. Besides water and substrate transport, AQPs are reported to be intrinsically involved in fiber development in cotton. Coincidentally, both cotton and flax fibres are cellulosic in nature. Thus, a cardinal role of AQPs in flax fibre development is envisaged. In flax, particularly PIP family members (PIP1-3, -4, -5; PIP2-1, PIP2-2, -4, -5, and -11) showed higher level of expression in stem during vegetative stage followed by green capsule stage indicating possible role of PIPs in bast fiber development. Targeted identification of the AQPs specifically involved in water equilibrium *vis-a-vis* fibre elongation will delineate the molecular mechanism of fibre development in flax. The AQPs identified in the present study provide wealth of information to be used for further characterization of aquaporins to understand their physiological role in this industrial cash crop.

## Methods

### Genome-wide identification and distribution of aquaporins in flax

The *Linum usitatissimum* v1.0 annotated scaffold assembly of flax genome was retrieved from phytozome database (https://phytozome.jgi.doe.gov/pz/portal.html). A local database of the protein sequences of flax genes was created in BioEdit ver. 7.2.5^74^. The 466 AQP genes from 10 different plant species^15^ were employed as query to identify putative orthologs of AQP genes in flax in local database using BLASTp. An e-value of 10^−5^ was kept as initial cut-off to identify high scoring pairs (HSPs). The blast output was tabulated and the HSPs showing >100 bit score were selected. Finally, redundant hits were removed to select unique sequences for further analysis.

### Structural characterizations of flax aquaporins

The AQP sequences retrieved from phytozome database were employed to identify respective genes from flax genome retrieved from GigaDB database (http://gigadb.org/) using local blast in BioEdit ver. 7.2.5^74^. The details of length and location of AQPs were obtained from phytozome database. The gene structure of flax AQPs was analyzed using GSDS ver. 2.0^75^.

### Identification of functional motif and transmembrane domains

The NPA motifs were identified in protein sequences using conserved domain database at NCBI (CDD). Transmembrane domains in the genes were identified using TMHMM and SOSUI software tools (http://www.cbs.dtu.dk, http://harrier.nagahama-i-bio.ac.jp). The transmembrane domains were further analysed manually to detect altered and/or missing domains.

### Phylogenetic analysis of flax AQPs

The AQP sequences were aligned using CLUSTALW alignment function in MEGA6^76^. The phylogenetic tree was constructed by using maximum likelihood method and the stability of the branch node was measured by performing 1000 bootstraps. The subfamilies PIP, SIP, TIP, NIP and XIPs were classified in accordance to the nomenclature used for arabidopsis, rice and poplar^9^,^33^. A phylogenetic tree was constructed using arabidopsis, rice, poplar and flax AQP sequences.

### Tertiary protein structure prediction

The protein sequences of LuTIP3 (TIP3-1, TIP3-2, TIP3-3, TIP3-4) and LuPIP2 (PIP2-4, PIP2-5, PIP2-7) were submitted to the Phyre2 protein-modeling server (www.sbg.bio.ic.ac.uk/*phyre2). The results obtained in the form of PDB files were uploaded to PoreWalker server to predict tertiary protein structure *vis-a-vis* pore size (http://www.ebi.ac.uk/thornton-srv/software/PoreWalker/) in identified TIPS and PIPs.

### Identification of major intrinsic protein coding orthologs in different *Linum* species

Raw RNA sequencing reads SRR957663, SRR957662, SRR957228 from *Linum bienne, Linum grandiflorum* and *L. leonii* respectively, were downloaded from Short Read Archive (SRA) in NCBI. The raw reads were examined for the adaptor sequences. The reads were used for *de novo* assembly using CLC Genomics Workbench (version 9.0; CLC bio, Aarhus, Denmark). Parameters used for the *de novo* assembly were: word size 20, automatic bubble size 20, and minimum contig length 200. The N50 contig value was determined by sorting all contigs in decreasing order of size and the contigs were added until the total size reached at least half of the total size of all assembled contigs. To map reads back to contigs, options as mismatch cost 2, insertion cost 3, deletion cost 3, length fraction 0.5, similarity fraction 0.8, and color space error cost 3 were provided. A local database of the assembled sequences of flax transcripts was created in BioEdit ver. 7.2.5^74^. The 51 AQP genes from flax were employed as query to identify putative orthologs of AQP genes in three different *Linum* species in local database using BLASTn. An e-value of 10^−5^ was kept as initial cut-off to identify high scoring pairs (HSPs). The blast output was tabulated and the HSPs showing >100 bit score were selected. Finally, redundant hits were removed to select unique sequences. Further, unique sequences having length of >290 bp were considered as *bona fide* AQP orthologs.

### Expression profiling of flax aquaporins

To identify the transcriptional evidence for the putative flax AQPs, their transcript sequence were used as query to search flax specific ESTs at the NCBI database (dated; Mar 2016; 2,86,856 sequences; http://blast.ncbi.nlm.nih.gov/) using BLASTn. The ESTs showing >99% identity were selected and the redundant hits were removed before determining number of EST hits for each AQP transcript.

The microarray data by Fenart, *et al*.^48^ was downloaded from NCBI GEO database (http://www.ncbi.nlm.nih.gov/geo/query/acc.cgi?acc=GSE21868). RMA normalized gene-level signal intensity (log2) values for the unigenes exhibiting similarity to AQPs were used from all replicates and averaged further. Similarly, the normalized RNA-Seq dataset generated by Kumar, *et al*.^77^ available at http://linum.ca/downloads/RNAseq was also used to analyze the expression of AQPs. A heat map for expression of AQPs was constructed with these values using TIGR Multi Experiment Viewer (MeV, http://www.tm4.org/mev.html). Hierarchical clustering with average linkage method was performed to cluster the genes.

Simultaneously, differential transcript expression data^49^ of apical region (AR) compared to basal region (BR) in flax (measured as normalized FPKM) was retrieved from NCBI GEO (GSE80718; http://www.ncbi.nlm.nih.gov/geo/query/acc.cgi?acc=GSE80718). The normalized FPKM values for flax MIPs identified in the present study were extracted. The MIPs showing at least two fold enrichment in AR compared to BR were selected.

Expression of TIP3 genes was analyzed across different tissues and developmental stages in, soybean, rice, arabidopsis, and *Medicago*. Absolute expression values were obtained from The Bio-Analytical Resource for Plant Biology (http://bbc.botany.utoronto.ca/) and converted into relative expression by comparing with the maximum level of expression in any given tissue.

### Silicon quantification in flax leaf and comparision of Si content in different plant species

Flax plants were grown in four replications with continuous supplementation of 1.7 mM Si in the form of potassium silicate as a regular irrigation. Leaf samples of 30 days-old plants were harvested and then dried at 65 °C for 24 h in hot air drier. The Si content in the dried powder was measured by HCL-HF extraction method followed by colorimetric analysis^78^. Phylogenetic tree of different plant species was developed based on the NCBI taxonomy using the PhytoT tool (http://phylot.biobyte.de/). Categorization of functional NIP2s with GSGR was based on attributes described in Deshmukh *et al*. 2015.

## Additional Information

**How to cite this article**: Shivaraj, S. M. *et al*. Genome-wide identification, characterization, and expression profile of aquaporin gene family in flax (*Linum usitatissimum*). *Sci. Rep.* **7**, 46137; doi: 10.1038/srep46137 (2017).

**Publisher's note:** Springer Nature remains neutral with regard to jurisdictional claims in published maps and institutional affiliations.

## Supplementary Material

Supplementary Information

## Figures and Tables

**Table 1:** Description and distribution of aquaporins identified from flax genome. Fifty one aquaporins belonging to five different classes such as PIPs(1–16), TIPs(17–33), NIPs(34–46), SIPs(47–49), and XIPs(49–51) along with their gene identifiers, gene length, and transcript length are identified in 43 scaffolds in flax genome.

Sl. No.	Gene	Phytozome ID	GigaDB ID	Gene Length (bp)	Trasncript Length (bp)	Protein length (aa)	Protein Molwt (kD)	Scaffold			No. of EST Hits
								Location	Start	End	
1	LuPIP1-1	Lus10014840	Lus_GLEAN_10016863	1142	912	303	32.65	scaffold 184	520725	521866	12
2	LuPIP1-2	Lus10028273	Lus_GLEAN_10031922	1202	864	287	30.72	scaffold 327	962214	963415	18
3	LuPIP1-3	Lus10035483	Lus_GLEAN_10039971	1647	864	287	30.58	scaffold 151	1507188	1508834	19
4	LuPIP1-4	Lus10024651	Lus_GLEAN_10027791	1954	864	287	30.64	scaffold 349	539057	541010	25
5	LuPIP1-5	Lus10032283	Lus_GLEAN_10036266	1866	864	287	30.64	scaffold 291	481896	483761	10
6	LuPIP2-1	Lus10027467	Lus_GLEAN_10031032	1662	849	282	29.89	scaffold 96	495844	497505	52
7	LuPIP2-2	Lus10014978	Lus_GLEAN_10017015	2228	849	282	29.99	scaffold 2022	333007	335234	24
8	LuPIP2-3	Lus10039222	Lus_GLEAN_10044037	1931	849	282	29.88	scaffold 33	47244	49174	37
9	LuPIP2-4	Lus10021475	Lus_GLEAN_10024185	4072	750	249	27.12	scaffold 612	628892	632963	5
10	LuPIP2-5	Lus10022577	Lus_GLEAN_10025492	847	594	197	21.28	scaffold 59	8029	8875	0
11	LuPIP2-6	Lus10023184	Lus_GLEAN_10026156	949	864	287	30.64	scaffold 325	843924	844872	17
12	LuPIP2-7	Lus10019934	Lus_GLEAN_10022468	1680	861	286	30.41	scaffold 1491	745462	747141	1
13	LuPIP2-8	Lus10026504	Lus_GLEAN_10029954	1427	600	199	21.12	scaffold 617	134836	136262	3
14	LuPIP2-9	Lus10041397	Lus_GLEAN_10046457	1170	798	265	27.99	scaffold 280	2244255	2245424	18
15	LuPIP2-10	Lus10023515	Lus_GLEAN_10026513	2270	876	291	30.75	scaffold 1216	608831	611100	0
16	LuPIP2-11	Lus10040399	Lus_GLEAN_10045377	2862	921	306	32.41	scaffold 86	1738335	1741196	1
17	LuTIP1-1	Lus10014411	Lus_GLEAN_10016362	991	759	252	26.06	scaffold 176	336786	337776	4
18	LuTIP1-2	Lus10023913	Lus_GLEAN_10026953	857	759	252	26.03	scaffold 177	490453	491309	10
19	LuTIP1-3	Lus10021510	Lus_GLEAN_10024223	857	759	252	25.78	scaffold 612	780365	781221	17
20	LuTIP1-4	Lus10022611	Lus_GLEAN_10025531	867	759	252	25.81	scaffold 59	169658	170524	13
21	LuTIP1-5	Lus10005885	Lus_GLEAN_10006756	939	759	252	25.63	scaffold 1158	163531	164469	1
22	LuTIP1-6	Lus10040863	Lus_GLEAN_10045876	939	759	252	25.63	scaffold 156	1895985	1896923	0
23	LuTIP1-7	Lus10003288	Lus_GLEAN_10003760	939	762	253	26.28	scaffold 762	27405	28343	0
24	LuTIP2-1	Lus10004733	Lus_GLEAN_10005415	1353	753	250	25.08	scaffold 2057	85764	87116	1
25	LuTIP2-2	Lus10007796	Lus_GLEAN_10008936	1381	753	250	25.09	scaffold 500	33724	35104	2
26	LuTIP2-3	Lus10025808	Lus_GLEAN_10029092	958	762	253	25.36	scaffold 605	486981	487938	3
27	LuTIP2-4	Lus10038293	Lus_GLEAN_10043013	930	747	248	24.85	scaffold 28	595621	596550	0
28	LuTIP3-1	Lus10018256	Lus_GLEAN_10020665	1070	771	256	27.16	scaffold 163	229180	230249	20
29	LuTIP3-2	Lus10040652	Lus_GLEAN_10045655	1413	771	256	27.30	scaffold 156	876812	878224	17
30	LuTIP3-3	Lus10036187	Lus_GLEAN_10040713	1275	774	257	27.35	scaffold 27	73940	75214	91
31	LuTIP3-4	Lus10038324	Lus_GLEAN_10043045	1387	774	257	27.37	scaffold 28	715833	717219	80
32	LuTIP4-1	Lus10037895	Lus_GLEAN_10042572	1769	744	247	25.79	scaffold 475	241334	243102	5
33	LuTIP5-1	Lus10031735	Lus_GLEAN_10035685	706	498	165	16.81	scaffold 783	450857	451562	0
34	LuNIP1-1	Lus10035999	Lus_GLEAN_10040510	1008	798	265	28.47	scaffold 76	651477	652484	0
35	LuNIP1-2	Lus10029274	Lus_GLEAN_10033012	1680	816	271	28.98	scaffold 360	400672	402351	0
36	LuNIP1-3	Lus10025744	Lus_GLEAN_10029024	974	879	292	30.88	scaffold 605	223796	224769	0
37	LuNIP1-4	Lus10035918	Lus_GLEAN_10040427	972	879	292	30.95	scaffold 76	270421	271392	0
38	LuNIP1-5	Lus10020929	Lus_GLEAN_10023548	1987	909	302	32.16	scaffold 711	673760	675746	0
39	LuNIP1-6	Lus10033447	Lus_GLEAN_10037660	2053	687	228	24.50	scaffold 701	17606	19658	0
40	LuNIP3-1	Lus10021935	Lus_GLEAN_10024751	1074	636	211	22.27	scaffold 164	655059	656132	0
41	LuNIP3-2	Lus10041222	Lus_GLEAN_10046267	1264	888	295	31.09	scaffold 280	1494684	1495947	0
42	LuNIP3-3	Lus10010153	Lus_GLEAN_10011538	3588	909	302	31.40	scaffold 587	43706	47293	0
43	LuNIP3-4	Lus10017358	Lus_GLEAN_10019645	2893	912	303	31.35	scaffold 511	252242	255134	0
44	LuNIP3-5	Lus10033268	Lus_GLEAN_10037463	6670	915	304	31.38	scaffold 488	326045	332714	1
45	LuNIP3-6	Lus10024066	Lus_GLEAN_10027115	1843	894	297	30.76	scaffold 353	358591	360433	0
46	LuNIP3-7	Lus10041674	Lus_GLEAN_10046750	1319	564	187	19.46	scaffold 272	1210252	1211570	1
47	LuSIP1-1	Lus10030046	Lus_GLEAN_10033849	735	735	244	25.64	scaffold 416	1226731	1227465	3
48	LuSIP1-2	Lus10035281	Lus_GLEAN_10039745	735	735	244	25.74	scaffold 151	29774	30508	0
49	LuXIP1-1	Lus10042385	Lus_GLEAN_10047501	1166	801	266	27.86	scaffold 123	1931033	1932198	11
50	LuXIP1-2	Lus10007568	Lus_GLEAN_10008664	4304	945	314	32.64	scaffold 259	161730	166033	0
51	LuXIP2-1	Lus10042375	Lus_GLEAN_10047491	1268	924	307	32.13	scaffold 123	1905398	1906665	0

**Table 2:** Conserved domains, selectivity filter and amino acid residues of AQPs in flax genome.

Loci	NPA (LB)	NPA (LE)	ar/R selectivity filters				Froger’s Residue				
			H2	H5	LE1	LE2	P1	P2	P3	P4	P5
**Plasma membrane intrinsic proteins (PIPs)**											
LuPIP1-1	NPA	NPA	F	H	T	R	Q	S	A	F	W
LuPIP1-2	NPA	NPA	F	H	T	R	E	S	A	F	W
LuPIP1-3	NPA	NPA	F	H	T	R	E	S	A	F	W
LuPIP1-4	NPA	NPA	F	H	T	R	E	S	A	F	W
LuPIP1-5	NPA	NPA	F	H	T	R	E	S	A	F	W
LuPIP2-1	NPA	NPA	F	H	T	R	M	S	A	F	W
LuPIP2-2	NPA	NPA	F	H	T	R	M	S	A	F	W
LuPIP2-3	NPA	NPA	F	H	T	R	M	S	A	F	W
LuPIP2-4	—	NPA	—	H	T	R	Q	S	A	F	W
LuPIP2-5	—	NPA	—	H	T	R	—	S	A	F	W
LuPIP2-6	NPA	NPA	F	H	T	R	Q	S	A	F	W
LuPIP2-7	NPA	NPA	F	H	T	R	Q	S	A	F	W
LuPIP2-8	NPA	NPA	—	H	T	R	Q	S	A	F	W
LuPIP2-9	GPA	NPA	F	H	T	R	Q	S	A	F	W
LuPIP2-10	NPA	NPA	F	H	T	R	Q	S	A	F	W
LuPIP2-11	NPA	NPA	F	H	T	R	Q	S	A	F	W
**Tonoplast intrinsic proteins (TIPs)**											
LuTIP1-1	NPA	NPA	H	I	A	V	T	S	A	Y	W
LuTIP1-2	NPA	NPA	H	I	A	V	T	S	A	Y	W
LuTIP1-3	NPA	NPA	H	I	A	V	T	S	A	Y	W
LuTIP1-4	NPA	NPA	H	I	A	V	T	S	A	Y	W
LuTIP1-5	NPA	NPA	H	I	A	V	T	S	A	Y	W
LuTIP1-6	PPA	NPA	H	I	A	V	T	S	A	Y	W
LuTIP1-7	NPA	NPA	H	I	A	L	T	S	A	Y	W
LuTIP2-1	NPA	NPA	H	I	G	R	T	S	A	Y	W
LuTIP2-2	NPA	NPA	H	I	G	R	T	S	A	Y	W
LuTIP2-3	NPA	NPA	H	I	G	R	T	S	A	Y	W
LuTIP2-4	NPA	NPA	H	I	G	R	T	S	A	Y	W
LuTIP3-1	NPA	NPA	H	I	A	R	T	A	S	Y	W
LuTIP3-2	NPA	NPA	H	I	A	R	T	A	T	Y	W
LuTIP3-3	NPA	NPA	H	I	A	R	T	A	S	Y	W
LuTIP3-4	NPA	NPA	H	I	A	R	T	A	S	Y	W
LuTIP4-1	NPA	NPA	H	I	A	R	T	S	A	Y	W
LuTIP5-1	—	NPA	—	V	G	C	T	A	A	Y	W
**Nodulin-26 like intrisic proteins (NIPs)**											
LuNIP1-1	NPA	NPA	W	V	A	R	F	S	A	Y	I
LuNIP1-2	NPA	NPA	W	V	A	R	F	S	A	Y	M
LuNIP1-3	NPA	NPA	W	V	A	R	F	S	A	Y	V
LuNIP1-4	NPA	NPA	W	V	A	R	F	S	A	Y	V
LuNIP1-5	NPA	NPA	W	V	A	R	F	S	A	Y	I
LuNIP1-6	—	NPA	—	V	A	R	F	S	A	Y	I
LuNIP3-1	NPA	NPA	—	V	G	R	Y	S	A	Y	I
LuNIP3-2	NPA	NPA	A	V	G	R	Y	S	A	Y	I
LuNIP3-3	NPA	NPV	S	I	A	R	F	A	A	Y	M
LuNIP3-4	NPA	NPV	S	I	A	R	F	A	A	Y	M
LuNIP3-5	NPA	NPV	S	I	A	R	F	T	A	Y	L
LuNIP3-6	NPA	NPV	T	I	A	R	F	T	A	Y	L
LuNIP3-7	NPA	NPV	—	I	A	R	F	T	A	Y	I
**Small basic intrinsic proteins (SIPs)**											
LuSIP1-1	NPT	NPA	A	I	P	N	R	A	A	Y	W
LuSIP1-2	NPT	NPA	A	I	P	N	R	A	A	Y	W
**Uncharacterized X intrinsic proteins (XIPs)**											
LuXIP1-1	NPV	NPA	I	L	V	R	V	C	A	F	W
LuXIP1-2	NPI	NPA	V	V	A	R	V	C	A	F	W
LuXIP2-1	NPV	NPA	I	V	V	R	M	C	A	F	W

**Table 3:** List of aquaporins identified in different *Linum* species. In comparison to 51 AQPs in *L. usitatissimum,* three related species of *Linum*such as *L. bienne* harbors 49 AQPs, *L. grandiflorum* posses 39 AQPs and *L. leonii* posses 19 AQPs.

Aquaporin Family	*L. usitatissimum*	*Linum bienne*	*Linum grandiflorum*	*Linum leonii*
NIP	LuNIP1-1	—	LgNIP1-1	—
	LuNIP1-2	LbNIP1-2a	LgNIP1-2a	LlNIP1-2
		LbNIP1-2b	LgNIP1-2b	—
	LuNIP3-1	—	—	—
	LuNIP3-2	LbNIP3-2a	LgNIP3-2	—
		LbNIP3-2b	—	—
	LuNIP3-3	LbNIP3-3	—	LlNIP3-3
	LuNIP3-4	—	LgNIP3-4	—
	LuNIP3-5	—	LgNIP3-5	LlNIP3-5
	LuNIP3-6	—	LgNIP3-6	—
	LuNIP3-7	—	—	—
	LuNIP1-3	—	—	—
	LuNIP1-4	—	—	—
	LuNIP1-5	—	—	—
	LuNIP1-6	—	—	—
PIP	LuPIP1-1	LbPIP1-1	LgPIP1-1	—
	LuPIP1-2	LbPIP1-2a	LgPIP1-2a	LlPIP1-2
		LbPIP1-2b	LgPIP1-2b	
	LuPIP1-3	LbPIP1-3a	LgPIP1-3	LlPIP1-3
		LbPIP1-3b	—	
	LuPIP1-4	LbPIP1-4a	LgPIP1-4	LlPIP1-4
		LbPIP1-4b	—	—
	LuPIP1-5	LbPIP1-5a	—	—
		LbPIP1-5b	—	—
	LuPIP2-1	—	—	LlPIP2-1
	LuPIP2-2	LbPIP2-2a	—	—
		LbPIP2-2b	—	—
	LuPIP2-3	LbPIP2-3	LgPIP2-3	LlPIP2-3
	LuPIP2-4	LbPIP2-4a	LgPIP2-4	—
		LbPIP2-4b	—	—
	LuPIP2-5	LbPIP2-5a	LgPIP2-5	LlPIP2-5
		LbPIP2-5b	—	
	LuPIP2-6	LbPIP2-6a	LgPIP2-6	LlPIP2-6a
		LbPIP2-6b	—	LlPIP2-6b
	LuPIP2-7	LbPIP2-7a	LgPIP2-7a	—
		LbPIP2-7b	LgPIP2-7b	—
		LbPIP2-7c	—	—
	LuPIP2-8	LbPIP2-8	—	—
	LuPIP2-9	LbPIP2-9a	LgPIP2-9a	LlPIP2-9
Aquaporin Family		LbPIP2-9b	LgPIP2-9b	—
		LbPIP2-9c	—	—
		LbPIP2-9d	—	—
	LuPIP2-10	LbPIP2-10	LgPIP2-10	—
	LuPIP2-11	LbPIP2-11a	LgPIP2-11	LlPIP2-11a
		LbPIP2-11b	—	LlPIP2-11b
TIP	LuTIP1-1	LbTIP1-1	LgTIP1-1	—
	LuTIP1-2	LbTIP1-2a	LgTIP1-2a	LlTIP1-2
		LbTIP1-2b	LgTIP1-2b	—
	LuTIP1-3	LbTIP1-3	LgTIP1-3	—
	LuTIP1-4	LbTIP1-4	LgTIP1-4	—
	LuTIP1-5	LbTIP1-5	LgTIP1-5	LlTIP1-5
	LuTIP1-6	LbTIP1-6	—	—
	LuTIP1-7	—	LgTIP1-7	—
	LuTIP2-1	—	—	—
	LuTIP2-2	LbTIP2-2	LgTIP2-2	LlTIP2-2
	LuTIP2-3	—	LgTIP2-3	—
	LuTIP2-4	LbTIP2-4	LgTIP2-4	—
	LuTIP3-1	—	—	—
	LuTIP3-2	LbTIP3-2	—	—
	LuTIP3-3	—	LgTIP3-3	—
	LuTIP3-4	—	LgTIP3-4	—
	LuTIP4-1	LbTIP4-1	LgTIP4-1	—
	LuTIP5-1	LbTIP5-1	LgTIP5-1	—
SIP	LuSIP1-1	LbSIP1-1	LgSIP1-1	LlSIP1-1
	LuSIP1-2	LbSIP1-2	—	—
XIP	LuXIP1-1	LbXIP1-1	LgXIP1-1	LlXIP1-1
	LuXIP1-2	—	—	—
	LuXIP2-1	—	LgXIP2-1	—
Number of MIPs	51	49	39	19
