# Supplementary material for: Genome-wide identification, characterization, and expression profile of aquaporin gene family in flax (Linum usitatissimum)
Source: Sci Rep. 2017 Apr 27;7:46137. doi: 10.1038/srep46137 (PMC5406838; doi:10.1038/srep46137)
Supplement: Supplementary Information [file srep46137-s1.doc]

**Supplementary information**

**Genome-wide identification, characterization, and expression profile of aquaporin gene family in Flax (*Linum usitatissimum*)**

S. M. Shivaraj^1,#^, Rupesh K Deshmukh^2,#^, Rhitu Rai^1,#^, Richard Belanger^2^, Pawan K. Agrawal^3^, Prasanta K Dash^1*^

*ICAR-NRC on Plant Biotechnology, PUSA, New Delhi, India*.

* Correspondence and requests for materials should be addressed to P.K.D. (email: pdas@nrcpb.org).

**Supplementary Table S1.** Details of Blastp results representing hits showing highest bit score for the respective queries from other plant genome.

| **Sl. No.** | **Query^#^** | **Blast Hit** | **e-value** | **Score (bit)** |
| --- | --- | --- | --- | --- |
| 1 | GhPIP1-11 | Lus10014840 | 4.00E-134 | 474 |
| 2 | BrPIP2-12 | Lus10014978 | 5.00E-148 | 520 |
| 3 | PtPIP2-5 | Lus10019934 | 1.00E-148 | 522 |
| 4 | BrPIP2-6 | Lus10021475 | 2.00E-79 | 292 |
| 5 | BrPIP2-6 | Lus10022577 | 8.00E-74 | 273 |
| 6 | PtPIP2-5 | Lus10023184 | 5.00E-147 | 517 |
| 7 | GmPIP2-3 | Lus10023515 | 3.00E-139 | 491 |
| 8 | GhPIP1-3 | Lus10024651 | 2.00E-153 | 538 |
| 9 | PtPIP2-6 | Lus10026504 | 9.00E-106 | 380 |
| 10 | BrPIP2-12 | Lus10027467 | 6.00E-149 | 523 |
| 11 | PtPIP1-1 | Lus10028273 | 8.00E-155 | 543 |
| 12 | GhPIP1-3 | Lus10032283 | 2.00E-153 | 538 |
| 13 | GhPIP1-3 | Lus10035483 | 3.00E-150 | 528 |
| 14 | BrPIP2-12 | Lus10039222 | 8.00E-149 | 523 |
| 15 | GmPIP2-3 | Lus10040399 | 3.00E-136 | 481 |
| 16 | PtPIP2-3 | Lus10041397 | 2.00E-122 | 435 |
| 17 | GmTIP1-9 | Lus10003288 | 2.00E-113 | 405 |
| 18 | SlTIP2-3 | Lus10004733 | 2.00E-124 | 441 |
| 19 | PtTIP1-1 | Lus10005885 | 7.00E-125 | 443 |
| 20 | SlTIP2-3 | Lus10007796 | 2.00E-123 | 438 |
| 21 | PtTIP1-6 | Lus10014411 | 8.00E-131 | 462 |
| 22 | PtTIP3-2 | Lus10018256 | 2.00E-123 | 438 |
| 23 | PtTIP1-5 | Lus10021510 | 9.00E-128 | 452 |
| 24 | PtTIP1-5 | Lus10022611 | 5.00E-129 | 457 |
| 25 | PtTIP1-6 | Lus10023913 | 2.00E-130 | 461 |
| 26 | GhTIP2-3 | Lus10025808 | 4.00E-120 | 427 |
| 27 | PtTIP5-2 | Lus10031735 | 1.00E-66 | 249 |
| 28 | PtTIP3-2 | Lus10036187 | 2.00E-128 | 455 |
| 29 | PtTIP4-1 | Lus10037895 | 3.00E-114 | 407 |
| 30 | GhTIP2-3 | Lus10038293 | 7.00E-122 | 433 |
| 31 | PtTIP3-2 | Lus10038324 | 5.00E-128 | 453 |
| 32 | PtTIP3-2 | Lus10040652 | 7.00E-123 | 436 |
| 33 | PtTIP1-1 | Lus10040863 | 8.00E-123 | 436 |
| 34 | PtNIP3-3 | Lus10010153 | 1.00E-138 | 489 |
| 35 | PtNIP3-3 | Lus10017358 | 2.00E-135 | 478 |
| 36 | PtNIP1-5 | Lus10020929 | 2.00E-91 | 332 |
| 37 | PtNIP3-5 | Lus10021935 | 8.00E-67 | 250 |
| 38 | PtNIP3-1 | Lus10024066 | 2.00E-130 | 462 |
| 39 | PtNIP1-5 | Lus10025744 | 4.00E-62 | 234 |
| 40 | PtNIP1-2 | Lus10029274 | 1.00E-127 | 452 |
| 41 | PtNIP3-4 | Lus10033268 | 1.00E-133 | 473 |
| 42 | PtNIP1-5 | Lus10033447 | 8.00E-53 | 204 |
| **Sl. No.** | **Query** | **Blast Hit** | **e-value** | **Score (bit)** |
| 43 | PtNIP1-5 | Lus10035918 | 2.00E-61 | 233 |
| 44 | PtNIP1-3 | Lus10035999 | 4.00E-107 | 384 |
| 45 | PtNIP3-5 | Lus10041222 | 4.00E-98 | 354 |
| 46 | PtNIP3-2 | Lus10041674 | 1.00E-87 | 320 |
| 47 | PtSIP1-3a | Lus10030046 | 9.00E-84 | 306 |
| 48 | PtSIP1-4 | Lus10035281 | 1.00E-82 | 302 |
| 49 | PtXIP1-3 | Lus10007568 | 2.00E-112 | 402 |
| 50 | GmXIP1-1 | Lus10042375 | 6.00E-69 | 258 |
| 51 | GhXIP1-1 | Lus10042385 | 3.00E-108 | 388 |

^#^Gm- *Glycine max*, Pt- *Populus trichocarpa*, At- *Arabidopsis thaliana*, Br- *Brassica rapa*, Gh- *Gossypium hirsutum*, Sl- *Solanum lycopersicum*

**Supplementary Table S2. Conserved domain analysis of AQPs identified from flax using CDD tool from NCBI.**

| **Query** | **PSSM-ID** | **E-Value** | **Bitscore** | **Accession** | **Short name** | **Superfamily** |
| --- | --- | --- | --- | --- | --- | --- |
| LuPIP1-1 | 278651 | 6.03E-93 | 273.424 | pfam00230 | MIP | cl00200 |
| LuPIP1-2 | 278651 | 3.92E-99 | 288.832 | pfam00230 | MIP | cl00200 |
| LuPIP1-3 | 278651 | 3.79E-92 | 271.113 | pfam00230 | MIP | cl00200 |
| LuPIP1-4 | 278651 | 1.37E-94 | 277.276 | pfam00230 | MIP | cl00200 |
| LuPIP1-5 | 278651 | 1.37E-94 | 277.276 | pfam00230 | MIP | cl00200 |
| LuPIP2-1 | 278651 | 2.48E-94 | 276.121 | pfam00230 | MIP | cl00200 |
| LuPIP2-2 | 278651 | 8.57E-97 | 282.669 | pfam00230 | MIP | cl00200 |
| LuPIP2-3 | 278651 | 2.80E-94 | 276.121 | pfam00230 | MIP | cl00200 |
| LuPIP2-4 | 294134 | 3.20E-57 | 180.591 | cl00200 | MIP superfamily | - |
| LuPIP2-5 | 294134 | 1.87E-46 | 150.931 | cl00200 | MIP superfamily | - |
| LuPIP2-6 | 278651 | 4.20E-97 | 283.439 | pfam00230 | MIP | cl00200 |
| LuPIP2-7 | 278651 | 5.70E-95 | 278.047 | pfam00230 | MIP | cl00200 |
| LuPIP2-8 | 294134 | 1.37E-73 | 220.267 | cl00200 | MIP superfamily | - |
| LuPIP2-9 | 294134 | 2.61E-64 | 199.081 | cl00200 | MIP superfamily | - |
| LuPIP2-10 | 278651 | 2.37E-94 | 276.891 | pfam00230 | MIP | cl00200 |
| LuPIP2-11 | 278651 | 5.74E-91 | 268.802 | pfam00230 | MIP | cl00200 |
| LuTIP1-1 | 177664 | 7.72E-144 | 401.472 | PLN00027 | PLN00027 | cl00200 |
| LuTIP1-2 | 177664 | 3.52E-147 | 409.946 | PLN00027 | PLN00027 | cl00200 |
| LuTIP1-3 | 177664 | 2.57E-155 | 430.747 | PLN00027 | PLN00027 | cl00200 |
| LuTIP1-4 | 177664 | 1.45E-155 | 431.132 | PLN00027 | PLN00027 | cl00200 |
| LuTIP1-5 | 177664 | 7.87E-142 | 396.464 | PLN00027 | PLN00027 | cl00200 |
| LuTIP1-6 | 177664 | 1.17E-140 | 393.383 | PLN00027 | PLN00027 | cl00200 |
| LuTIP1-7 | 177664 | 6.62E-139 | 389.145 | PLN00027 | PLN00027 | cl00200 |
| LuTIP2-1 | 294134 | 6.43E-122 | 346.154 | cl00200 | MIP superfamily | - |
| LuTIP2-2 | 294134 | 5.81E-121 | 343.457 | cl00200 | MIP superfamily | - |
| LuTIP2-3 | 294134 | 2.02E-113 | 324.583 | cl00200 | MIP superfamily | - |
| LuTIP2-4 | 294134 | 4.70E-117 | 333.442 | cl00200 | MIP superfamily | - |
| LuTIP4-1 | 294134 | 2.58E-85 | 253.17 | cl00200 | MIP superfamily | - |
| LuTIP3-1 | 294134 | 2.37E-101 | 294.001 | cl00200 | MIP superfamily | - |
| LuTIP3-2 | 294134 | 1.68E-100 | 292.075 | cl00200 | MIP superfamily | - |
| LuTIP3-3 | 294134 | 1.40E-100 | 292.075 | cl00200 | MIP superfamily | - |
| LuTIP3-4 | 294134 | 1.45E-100 | 292.075 | cl00200 | MIP superfamily | - |
| LuTIP5-1 | 294134 | 2.97E-75 | 224.397 | cl00200 | MIP superfamily | - |
| LuNIP1-1 | 294134 | 4.99E-127 | 360.764 | cl00200 | MIP superfamily | - |
| LuNIP1-2 | 294134 | 1.47E-133 | 378.21 | cl00200 | MIP superfamily | - |
| LuNIP1-3 | 294134 | 1.04E-65 | 205.913 | cl00200 | MIP superfamily | - |
| LuNIP1-4 | 294134 | 2.67E-65 | 204.758 | cl00200 | MIP superfamily | - |
| LuNIP1-5 | 294134 | 7.56E-73 | 224.9 | cl00200 | MIP superfamily | - |
| LuNIP1-6 | 294134 | 9.21E-43 | 145.164 | cl00200 | MIP superfamily | - |
| **Query** | **PSSM-ID** | **E-Value** | **Bitscore** | **Accession** | **Short name** | **Superfamily** |
| LuNIP3-1 | 294134 | 5.42E-86 | 254.011 | cl00200 | MIP superfamily | - |
| LuNIP3-2 | 294134 | 1.55E-124 | 355.318 | cl00200 | MIP superfamily | - |
| LuNIP3-3 | 177663 | 0 | 499.389 | PLN00026 | PLN00026 | cl00200 |
| LuNIP3-4 | 177663 | 3.79E-176 | 487.447 | PLN00026 | PLN00026 | cl00200 |
| LuNIP3-5 | 177663 | 4.37E-179 | 494.766 | PLN00026 | PLN00026 | cl00200 |
| LuNIP3-6 | 177663 | 7.00E-156 | 435.831 | PLN00026 | PLN00026 | cl00200 |
| LuNIP3-7 | 177663 | 4.71E-109 | 312.567 | PLN00026 | PLN00026 | cl00200 |
| LuSIP1-1 | 294134 | 4.21E-10 | 56.493 | cl00200 | MIP superfamily | - |
| LuSIP1-2 | 294134 | 5.92E-09 | 53.4114 | cl00200 | MIP superfamily | - |
| LuXIP1-1 | 294134 | 4.07E-33 | 119.281 | cl00200 | MIP superfamily | - |
| LuXIP1-2 | 294134 | 9.60E-33 | 119.666 | cl00200 | MIP superfamily | - |
| LuXIP2-1 | 294134 | 1.39E-31 | 116.263 | cl00200 | MIP superfamily | - |

**Supplementary Table S3. Functional annotation of AQPs identified from flax using BLAST2GO.**

| **Gene ID** | **Hit**  **Description** | **e-Value** | **sim mean** | **#Gos** | | | **InterPro IDs** | |
| --- | --- | --- | --- | --- | --- | --- | --- | --- |
| Lus10003288 | Aquaporin TIP1-3 | 3.00E-127 | 88% | | 6 | PTHR19139:SF151; IPR000425 (PANTHER) | |  |
| Lus10004733 | Probable aquaporin TIP-type | 4.10E-118 | 91% | | 4 | IPR000425; PTHR19139:SF134 (PANTHER) | |  |
| Lus10005885 | Aquaporin TIP1-3 | 2.70E-127 | 90% | | 6 | IPR000425; PTHR19139:SF55 (PANTHER) | |  |
| Lus10007568 | Aquaporin PIP1-1 Short | 3.20E-23 | 48% | | 3 | IPR000425; PTHR19139:SF162 (PANTHER) | |  |
| Lus10007796 | Probable aquaporin TIP-type | 8.90E-117 | 90% | | 4 | PTHR19139:SF134; IPR000425 (PANTHER) | |  |
| Lus10010153 | Probable aquaporin NIP5-1 | 5.50E-141 | 87% | | 9 | IPR000425; PTHR19139:SF102 (PANTHER) | |  |
| Lus10014411 | Probable aquaporin TIP1-1 | 2.70E-120 | 89% | | 8 | PTHR19139:SF52; IPR000425 (PANTHER) | |  |
| Lus10014840 | Aquaporin PIP1-5 | 5.80E-154 | 86% | | 4 | IPR000425; PTHR19139:SF90 (PANTHER) | |  |
| Lus10014978 | Aquaporin PIP2-4 | 8.10E-152 | 82% | | 9 | PTHR19139:SF152; IPR000425 (PANTHER) | |  |
| Lus10017358 | Probable aquaporin NIP5-1 | 2.20E-149 | 87% | | 9 | IPR000425; PTHR19139:SF102 (PANTHER) | |  |
| Lus10018256 | Probable aquaporin TIP3-2 | 4.30E-127 | 88% | | 4 | IPR000425; PTHR19139:SF30 (PANTHER) | |  |
| Lus10019934 | Aquaporin PIP2-4 | 1.30E-173 | 89% | | 4 | PTHR19139:SF167; IPR000425 (PANTHER) | |  |
| Lus10020929 | Nodulin-26 Short | 2.20E-72 | 71% | | 1 | PTHR19139:SF145; IPR000425 (PANTHER) | |  |
| Lus10021475 | Aquaporin PIP2-4 | 5.70E-87 | 76% | | 1 | PTHR19139:SF158; IPR000425 (PANTHER) | |  |
| Lus10021510 | Aquaporin TIP1-1 | 6.00E-141 | 88% | | 11 | IPR000425; PTHR19139:SF52 (PANTHER) | |  |
| Lus10021935 | Probable aquaporin NIP7-1 | 9.80E-50 | 79% | | 4 | PTHR19139:SF171; IPR000425 (PANTHER) | |  |
| Lus10022577 | Aquaporin PIP2-1 | 6.90E-82 | 79% | | 4 | PTHR19139:SF158; IPR000425 (PANTHER) | |  |
| Lus10022611 | Aquaporin TIP1-1 | 3.50E-141 | 87% | | 11 | IPR000425; PTHR19139:SF52 (PANTHER) | |  |
| Lus10023184 | Aquaporin PIP2-2 | 2.40E-174 | 89% | | 8 | IPR000425; PTHR19139:SF167 (PANTHER) | |  |
| Lus10023515 | Aquaporin PIP2-5 | 3.30E-153 | 87% | | 4 | IPR000425; PTHR19139:SF158 (PANTHER) | |  |
| Lus10023913 | Probable aquaporin TIP1-1 | 1.70E-118 | 88% | | 8 | PTHR19139:SF52; IPR000425 (PANTHER) | |  |
|  |  |  |  | |  |  | |  |
| **Gene ID** | **Hit**  **Description** | **e-Value** | **sim mean** | | **#Gos** | **InterPro IDs** | |  |
| Lus10024066 | Aquaporin NIP6-1 | 3.20E-129 | 83% | | 9 | PTHR19139:SF170; IPR000425 (PANTHER) | |  |
| Lus10024651 | Aquaporin PIP1-2 Short | 0.00E+00 | 95% | | 11 | IPR000425; PTHR19139:SF169 (PANTHER) | |  |
| Lus10025744 | Nodulin-26 Short | 1.00E-66 | 64% | | 1 | IPR000425; PTHR19139:SF145 (PANTHER) | |  |
| Lus10025808 | Aquaporin TIP2-1 | 1.50E-97 | 90% | | 15 | PTHR19139:SF99; IPR000425 (PANTHER) | |  |
| Lus10026504 | Aquaporin PIP2-1 | 3.20E-126 | 95% | | 4 | PTHR19139:SF158; IPR000425 (PANTHER) | |  |
| Lus10027467 | Probable aquaporin PIP2-8 | 0.00E+00 | 93% | | 7 | IPR000425; PTHR19139:SF152 (PANTHER) | |  |
| Lus10028273 | Probable aquaporin PIP1-2 | 0.00E+00 | 93% | | 9 | IPR000425; PTHR19139:SF146 (PANTHER) | |  |
| Lus10029274 | Aquaporin NIP1-2 | 3.60E-132 | 79% | | 9 | IPR000425; PTHR19139:SF144 (PANTHER) | |  |
| Lus10030046 | Aquaporin SIP1-1 | 1.60E-57 | 70% | | 7 | IPR000425; PTHR19139:SF75 (PANTHER) | |  |
| Lus10031735 | Probable aquaporin TIP5-1 | 5.80E-59 | 75% | | 8 | IPR000425; PTHR19139:SF54 (PANTHER) | |  |
| Lus10032283 | Aquaporin PIP1-2 Short | 0.00E+00 | 95% | | 11 | IPR000425; PTHR19139:SF169 (PANTHER) | |  |
| Lus10033268 | Probable aquaporin NIP5-1 | 7.50E-138 | 87% | | 9 | IPR000425; PTHR19139:SF102 (PANTHER) | |  |
| Lus10033447 | Nodulin-26 Short | 4.80E-42 | 73% | | 1 | PTHR19139:SF145; IPR000425 (PANTHER) | |  |
| Lus10035281 | Aquaporin SIP1-2 | 2.20E-62 | 71% | | 4 | PTHR19139:SF75; IPR000425 (PANTHER) | |  |
| Lus10035483 | Aquaporin PIP1-2 Short | 0.00E+00 | 94% | | 11 | PTHR19139:SF169; IPR000425 (PANTHER) | |  |
| Lus10035918 | Aquaporin NIP1-1 | 1.00E-63 | 68% | | 6 | PTHR19139:SF145; IPR000425 (PANTHER) | |  |
| Lus10035999 | Probable aquaporin NIP-type | 1.70E-126 | 80% | | 3 | PTHR19139:SF181; IPR000425 (PANTHER) | |  |
| Lus10036187 | Aquaporin TIP3-1 | 3.80E-130 | 87% | | 7 | IPR000425; PTHR19139:SF30 (PANTHER) | |  |
| Lus10037895 | Aquaporin TIP4-1 | 2.50E-112 | 87% | | 5 | IPR000425; PTHR19139:SF143 (PANTHER) | |  |
| Lus10038293 | Aquaporin TIP2-1 | 2.10E-100 | 91% | | 15 | IPR000425; PTHR19139:SF99 (PANTHER) | |  |
| Lus10038324 | Aquaporin TIP3-1 | 1.40E-129 | 87% | | 7 | IPR000425; PTHR19139:SF30 (PANTHER) | |  |
|  |  |  |  | |  |  | |  |
|  |  |  |  | |  |  | |  |
| **Gene ID** | **Hit**  **Description** | **e-Value** | **sim mean** | | **#Gos** | **InterPro IDs** | |  |
| Lus10039222 | Probable aquaporin PIP2-8 | 0.00E+00 | 93% | | 7 | IPR000425; PTHR19139:SF152 (PANTHER) | |  |
| Lus10040399 | Aquaporin PIP2-2 | 1.50E-150 | 82% | | 4 | IPR000425; PTHR19139:SF158 (PANTHER) | |  |
| Lus10040652 | Probable aquaporin TIP3-2 | 5.60E-126 | 87% | | 4 | IPR000425; PTHR19139:SF30 (PANTHER) | |  |
| Lus10040863 | Aquaporin TIP1-3 | 7.60E-115 | 89% | | 6 | IPR000425; PTHR19139:SF55 (PANTHER) | |  |
| Lus10041222 | Probable aquaporin NIP7-1 | 3.10E-79 | 75% | | 4 | IPR000425; PTHR19139:SF171 (PANTHER) | |  |
| Lus10041397 | Aquaporin PIP2-1 | 1.60E-137 | 79% | | 11 | PTHR19139:SF167; IPR000425 (PANTHER) | |  |
| Lus10041674 | Aquaporin NIP6-1 | 4.60E-85 | 83% | | 9 | PTHR19139:SF170; IPR000425 (PANTHER) | |  |
| Lus10042375 | Aquaporin TIP3-1 | 1.10E-22 | 48% | | 2 | IPR000425; PTHR19139:SF172 (PANTHER) | |  |
| Lus10042385 | Aquaporin TIP4-2 | 2.40E-13 | 49% | | 6 | PTHR19139:SF162; IPR000425 (PANTHER) | |  |

**Supplementary Table S4. Transmembrane domains in AQPs identified from flax using TMHMM and SOSUI servers.**

|  |  |  | **TMHMM** |  |  | **SOSUI** |  |  |
| --- | --- | --- | --- | --- | --- | --- | --- | --- |
| **gene** | **Length** | **ExpAA** | **First 60** | **predHel** |  | **protein type** | **helix** | **Max TM** |
| LuPIP1-1 | 303 | 130.9 | 0.03 | 6 |  | MP | 5 | 6 |
| LuPIP1-2 | 287 | 127.1 | 4.92 | 6 |  | MP | 5 | 6 |
| LuPIP1-3 | 287 | 130.69 | 6.74 | 6 |  | MP | 6 | 6 |
| LuPIP1-4 | 287 | 128.42 | 6.99 | 6 |  | MP | 6 | 6 |
| LuPIP1-5 | 287 | 128.42 | 6.99 | 6 |  | MP | 6 | 6 |
| LuPIP2-1 | 282 | 135.38 | 20.39 | 6 |  | MP | 5 | 6 |
| LuPIP2-2 | 282 | 135.5 | 20.73 | 6 |  | MP | 5 | 6 |
| LuPIP2-3 | 282 | 135.4 | 20.43 | 6 |  | MP | 5 | 6 |
| LuPIP2-4^#^ | 249 | 85.51 | 0 | 4 |  | MP | 3 | 4 |
| LuPIP2-5^#^ | 197 | 85.97 | 18.67 | 4 |  | MP | 3 | 4 |
| LuPIP2-6 | 287 | 130.74 | 21.47 | 6 |  | MP | 5 | 6 |
| LuPIP2-7 | 286 | 130.11 | 19.81 | 6 |  | MP | 5 | 6 |
| LuPIP2-8^#^ | 199 | 103.94 | 42.32 | 5 |  | MP | 3 | 5 |
| LuPIP2-9^#^ | 265 | 132.84 | 20.98 | 6 |  | MP | 5 | 6 |
| LuPIP2-10 | 291 | 134.5 | 21.62 | 6 |  | MP | 5 | 6 |
| LuPIP2-11 | 306 | 149.76 | 21.7 | 7 |  | MP | 5 | 7 |
| LuTIP1-1 | 252 | 141.01 | 24.25 | 6 |  | MP | 6 | 6 |
| LuTIP1-2 | 252 | 142.95 | 23.02 | 6 |  | MP | 6 | 6 |
| LuTIP1-3 | 252 | 139.75 | 23.22 | 6 |  | MP | 6 | 6 |
| LuTIP1-4 | 252 | 140.43 | 23.49 | 6 |  | MP | 6 | 6 |
| LuTIP1-5 | 252 | 143.17 | 25.07 | 6 |  | MP | 6 | 6 |
| LuTIP1-6 | 252 | 143.36 | 25.32 | 6 |  | MP | 6 | 6 |
| LuTIP1-7 | 253 | 139.65 | 25.84 | 7 |  | MP | **5** | 7 |
| LuTIP2-1 | 250 | 151.11 | 29.18 | 7 |  | MP | 6 | 7 |
| LuTIP2-2 | 250 | 155.15 | 29.43 | 7 |  | MP | 7 | 7 |
| LuTIP2-3 | 253 | 151.83 | 29.39 | 6 |  | MP | 6 | 6 |
| LuTIP2-4 | 248 | 152.52 | 29.45 | 7 |  | MP | 6 | 7 |
| LuTIP3-1 | 256 | 132.59 | 25.15 | 6 |  | MP | 6 | 6 |
| LuTIP3-2 | 256 | 132.38 | 24.11 | 6 |  | MP | 6 | 6 |
| LuTIP3-3 | 257 | 134.05 | 25.8 | 6 |  | MP | 6 | 6 |
| LuTIP3-4 | 257 | 133.63 | 25.83 | 6 |  | MP | 6 | 6 |
| LuTIP4-1 | 247 | 141.82 | 33.95 | 6 |  | MP | 6 | 6 |
| LuTIP5-1^#^ | 165 | 88.15 | 34.38 | 4 |  | MP | 4 | 4 |
| LuNIP1-1 | 265 | 131.43 | 20.17 | 6 |  | MP | 6 | 6 |
|  |  |  |  |  |  |  |  |  |
|  |  |  |  |  |  |  |  |  |
|  |  |  | **TMHMM** |  |  | **SOSUI** |  |  |
| **gene** | **Length** | **ExpAA** | **First 60** | **predHel** |  | **protein type** | **helix** | **Max TM** |
| LuNIP1-2 | 271 | 126.5 | 18.27 | 6 |  | MP | 5 | 6 |
| LuNIP1-3 | 292 | 116.26 | 0.14 | 6 |  | MP | 6 | 6 |
| LuNIP1-4 | 292 | 118.46 | 0.26 | 5 |  | MP | 6 | 6 |
| LuNIP1-5 | 302 | 121.07 | 0 | 5 |  | MP | 6 | 6 |
| LuNIP1-6^#^ | 228 | 84.92 | 0 | 4 |  | MP | 4 | 4 |
| LuNIP3-1^#^ | 211 | 90.79 | 5.3 | 4 |  | MP | 4 | 4 |
| LuNIP3-2 | 295 | 134.22 | 5.69 | 6 |  | MP | 6 | 6 |
| LuNIP3-3 | 302 | 127.93 | 0.06 | 6 |  | MP | 6 | 6 |
| LuNIP3-4 | 303 | 127.87 | 0.06 | 6 |  | MP | 6 | 6 |
| LuNIP3-5 | 304 | 123.81 | 0.01 | 5 |  | MP | 6 | 5 |
| LuNIP3-6 | 297 | 128.68 | 0 | 6 |  | MP | 6 | 6 |
| LuNIP3-7^#^ | 187 | 95.97 | 30.38 | 5 |  | MP | 4 | 5 |
| LuSIP1-1 | 244 | 111.07 | 39.05 | 5 |  | MP | 5 | 5 |
| LuSIP1-2 | 244 | 111.51 | 37.6 | 5 |  | MP | 6 | 6 |
| LuXIP1-2 | 314 | 149.6 | 0.01 | 7 |  | MP | 6 | 7 |
| LuXIP2-1 | 307 | 128.62 | 7.65 | 6 |  | MP | 6 | 6 |
| LuXIP1-1 | 266 | 145.31 | 26.78 | 7 |  | MP | 6 | 7 |

^#^ Sequences showing less than six transmembrane domains predicted based on their sequence alignment.

ExpAA: The expected number of amino acids in transmembrane helices.

First60: The expected number of amino acids in transmembrane helices in the first 60 amino acids of the protein.

MP: Membrane protein

TMH: The number of predicted transmembrane helices.

MAX TMH: The maximum number among the TMH predicted by TMHMM and SOSUI

**Supplementary Table S5.** Details of predicted sub-cellular location of flax AQPs identified by using Wolfpsort, Cello and TargetP servers.

| **Gene_ID** | **Wolfpsort** | **Cello** | **TargetP** |
| --- | --- | --- | --- |
| LuPIP1-1 | Plasma membrane | PlasmaMembrane | _ |
| LuPIP1-2 | Plasma membrane | PlasmaMembrane | _ |
| LuPIP1-3 | Plasma membrane | PlasmaMembrane | _ |
| LuPIP1-4 | Plasma membrane | PlasmaMembrane | _ |
| LuPIP1-5 | Plasma membrane | PlasmaMembrane | _ |
| LuPIP2-1 | Plasma membrane | PlasmaMembrane | _ |
| LuPIP2-2 | Plasma membrane | PlasmaMembrane | _ |
| LuPIP2-3 | Plasma membrane | PlasmaMembrane | _ |
| LuPIP2-4 | Plasma membrane | PlasmaMembrane | _ |
| LuPIP2-5 | Plasma membrane | PlasmaMembrane | _ |
| LuPIP2-6 | Plasma membrane | PlasmaMembrane | _ |
| LuPIP2-7 | Plasma membrane | PlasmaMembrane | _ |
| LuPIP2-8 | cytoplasm | PlasmaMembrane | _ |
| LuPIP2-9 | Plasma membrane | PlasmaMembrane | _ |
| LuPIP2-10 | Plasma membrane | PlasmaMembrane | _ |
| LuPIP2-11 | Plasma membrane | PlasmaMembrane | _ |
| LuTIP1-1 | vacuole | PlasmaMembrane | _ |
| LuTIP1-2 | vacuole | PlasmaMembrane | _ |
| LuTIP1-3 | vacuole | PlasmaMembrane | _ |
| LuTIP1-4 | vacuole | PlasmaMembrane | _ |
| LuTIP1-5 | cytoplasm | PlasmaMembrane | _ |
| LuTIP1-6 | cytoplasm | PlasmaMembrane | _ |
| LuTIP1-7 | vacuole | PlasmaMembrane | _ |
| LuTIP2-1 | Plasma membrane | PlasmaMembrane | Secretory |
| LuTIP2-2 | Plasma membrane | PlasmaMembrane | Secretory |
| LuTIP2-3 | cytoplasm | PlasmaMembrane | Secretory |
| LuTIP2-4 | cytoplasm | PlasmaMembrane | Secretory |
| LuTIP3-1 | cytoplasm | PlasmaMembrane | _ |
| LuTIP3-2 | cytoplasm | PlasmaMembrane | _ |
| LuTIP3-3 | cytoplasm | PlasmaMembrane | _ |
| LuTIP3-4 | cytoplasm | PlasmaMembrane | _ |
| LuTIP4-1 | cytoplasm | PlasmaMembrane | _ |
| LuTIP5-1 | chloroplast | PlasmaMembrane | Secretory |
| LuNIP1-1 | Plasma membrane | PlasmaMembrane | _ |
| LuNIP1-2 | Plasma membrane | PlasmaMembrane | _ |
| **Gene_ID** | **Wolfpsort** | **Cello** | **TargetP** |
| LuNIP1-3 | cytoplasm | PlasmaMembrane | _ |
| LuNIP1-4 | Plasma membrane | PlasmaMembrane | Chloroplast |
| LuNIP1-5 | cytoplasm | PlasmaMembrane | Chloroplast |
| LuNIP1-6 | cytoplasm | PlasmaMembrane | _ |
| LuNIP3-1 | chloroplast | PlasmaMembrane | _ |
| LuNIP3-2 | Plasma membrane | PlasmaMembrane | _ |
| LuNIP3-3 | Plasma membrane | PlasmaMembrane | Chloroplast |
| LuNIP3-4 | Plasma membrane | PlasmaMembrane | Chloroplast |
| LuNIP3-5 | Plasma membrane | PlasmaMembrane | _ |
| LuNIP3-6 | Plasma membrane | PlasmaMembrane | Chloroplast |
| LuNIP3-7 | vacuole | PlasmaMembrane | _ |
| LuSIP1-1 | vacuole | PlasmaMembrane | Secretory |
| LuSIP1-2 | vacuole | PlasmaMembrane | Secretory |
| LuXIP1-1 | Plasma membrane | PlasmaMembrane | _ |
| LuXIP1-2 | Plasma membrane | PlasmaMembrane | _ |
| LuXIP2-1 | cytoplasm | PlasmaMembrane | _ |

^1^http://wolfpsort.org/, ^2^http://cello.life.nctu.edu.tw, ^3^www.cbs.dtu.dk/services/TargetP

**Supplementary Table S6**. Summary of *de novo* sequence assembly of RNA reads from different *Linum* species.

|  | ***L. bienne*** | ***L. grandiflorum*** | ***L. leonii*** |
| --- | --- | --- | --- |
| N75 (bp) | 340 | 414 | 569 |
| N50 (bp) | 520 | 797 | 1254 |
| N25 (bp) | 779 | 1444 | 2103 |
| Minimum length (bp) | 161 | 172 | 153 |
| Maximum length (bp) | 4839 | 6069 | 13226 |
| Average length (bp) | 460 | 606 | 788 |
| Count | 46597 | 46333 | 42305 |

**Supplementary Table S7**. Normalized differential transcript abundance of different aquaporins between apical region and basal region in flax.

| **Gene Name** | **Gene ID** | **log2(fold change AR/BR)** |
| --- | --- | --- |
| LuPIP1-2 | Lus10028273 | 2.16375 |
| LuPIP2-2 | Lus10014978 | 2.73709 |
| LuPIP2-4 | Lus10021475 | 12.0318 |
| LuPIP2-5 | Lus10022577 | 11.0516 |
| LuPIP2-7 | Lus10019934 | 5.36766 |
| LuPIP2-8 | Lus10026504 | 3.41476 |
| LuPIP2-9 | Lus10041397 | 3.69459 |
| LuTIP1-1 | Lus10014411 | 3.20655 |
| LuTIP1-2 | Lus10023913 | 3.09236 |
| LuTIP1-6 | Lus10040863 | 3.27783 |
| LuTIP2-3 | Lus10025808 | 4.19286 |
| LuTIP2-4 | Lus10038293 | 3.67796 |
| LuNIP1-2 | Lus10029274 | 5.96075 |
| LuNIP3-3 | Lus10010153 | 4.34727 |
| LuNIP3-4 | Lus10017358 | 5.52775 |
| LuNIP3-5 | Lus10033268 | 6.60565 |
| LuNIP3-7 | Lus10041674 | 3.91799 |
| LuXIP1-1 | Lus10042385 | 2.64329 |

|  |
| --- |
| **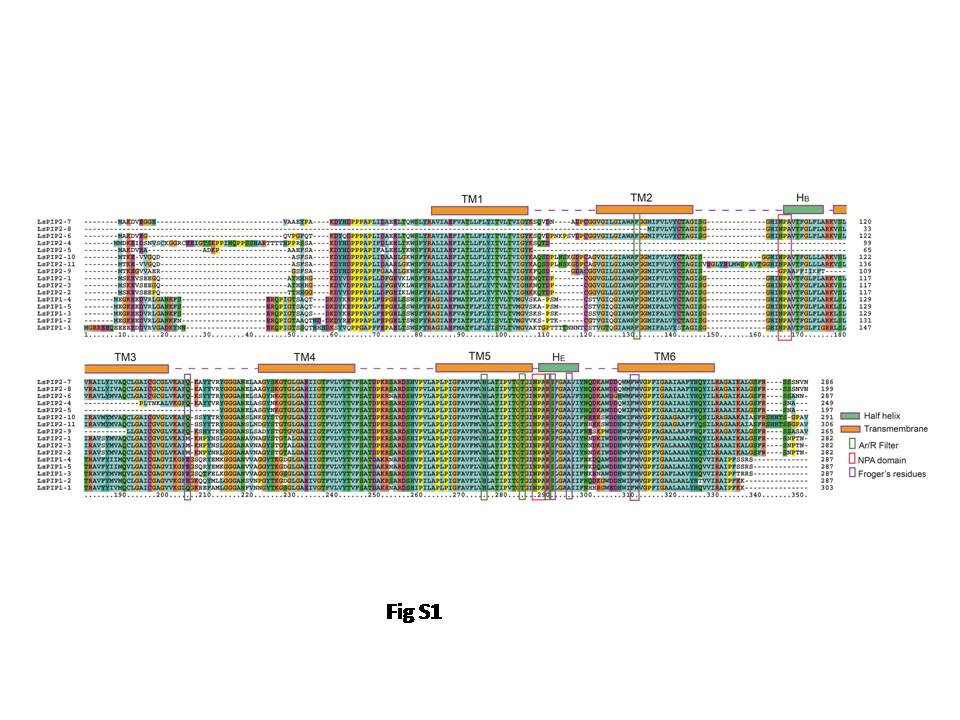** |

**Supplementary Figure S1.** Protein sequence alignment of PIPs identified in flax showing conserved transmembrane domains and amino acids at NPA domains, ar/R filters, Froger’s residues.

| 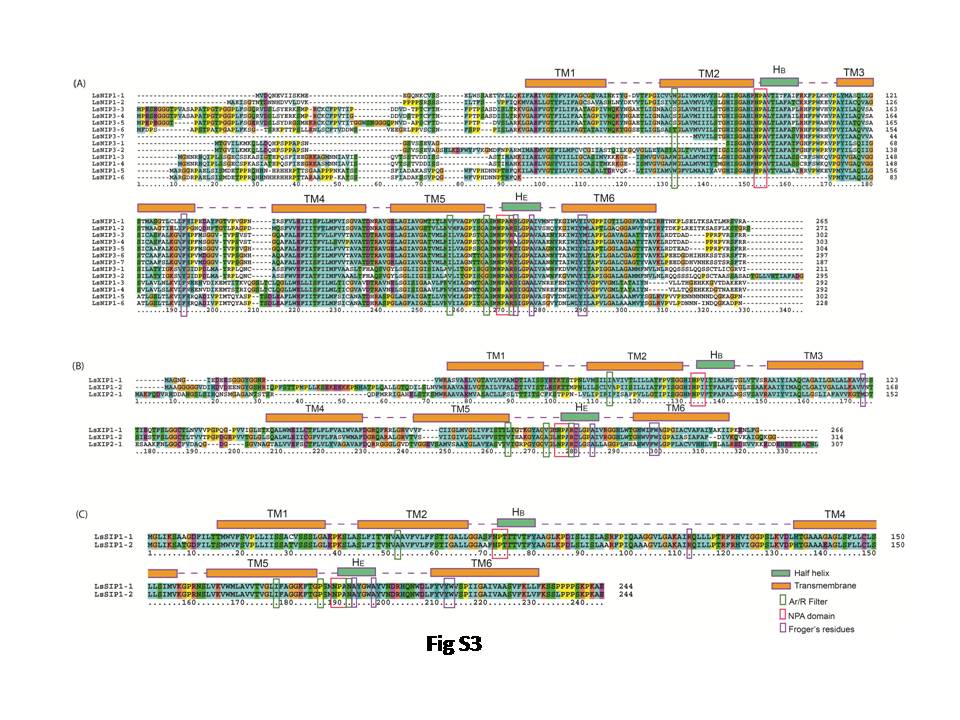 |
| --- |
|  |

**Supplementary Figure S2.** Protein sequence alignment of NIPs (A), SIPs (B) and XIPs (C) identified in flax showing conserved transmembrane domains and amino acids at NPA domains, ar/R filters, Froger’s residues.


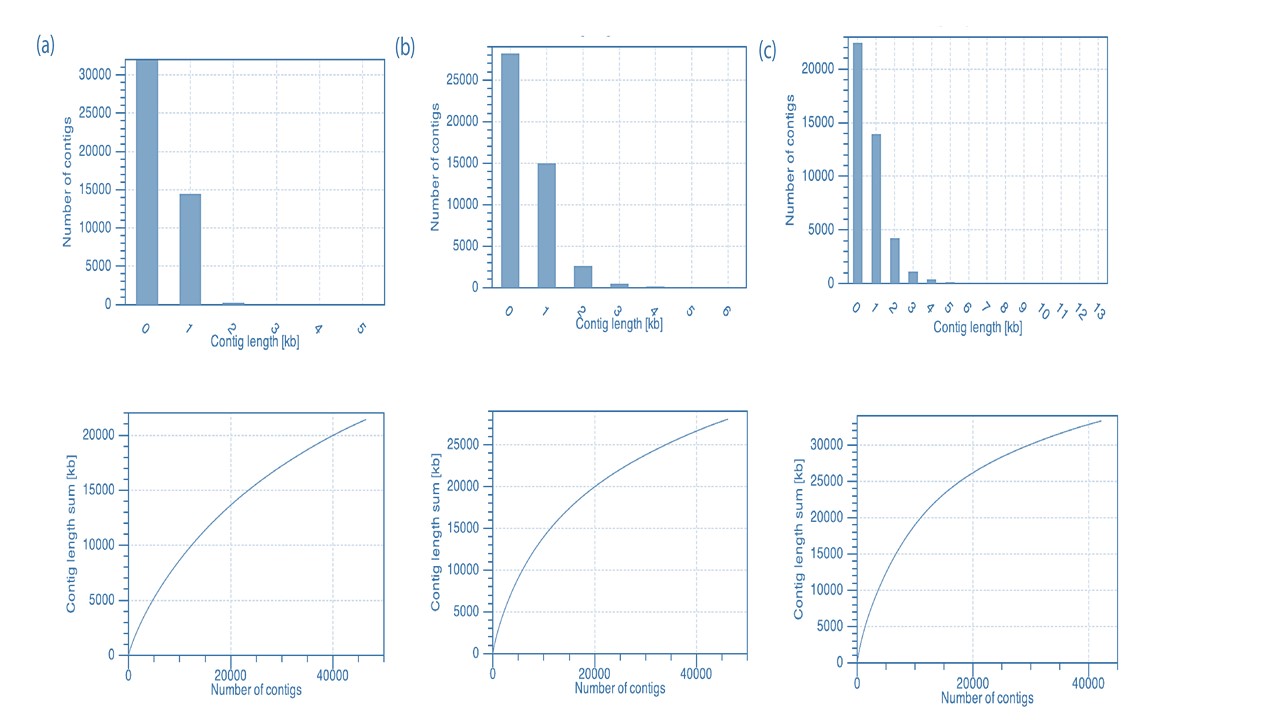
A)

**B)**

**Supplementary Figure S3.** (A) *de novo* assembly of RNA sequence reads showing number of contigs and contig length distribution in *L. bienne*(a), *L. grandiflorum* (b) and *L. leonii* (c). (B) Phylogenetic tree of Aquaporins (AQPs) identified in *L. bienne* (Lb), *L. grandiflorum* (Lg) and *L. leonii* (Ll) along with Flax (Lu). The tree was build using the DNA sequence alignment of AQP homologs created using ClustalW and maximum likelihood method provided in MEGA6 software tool.

**
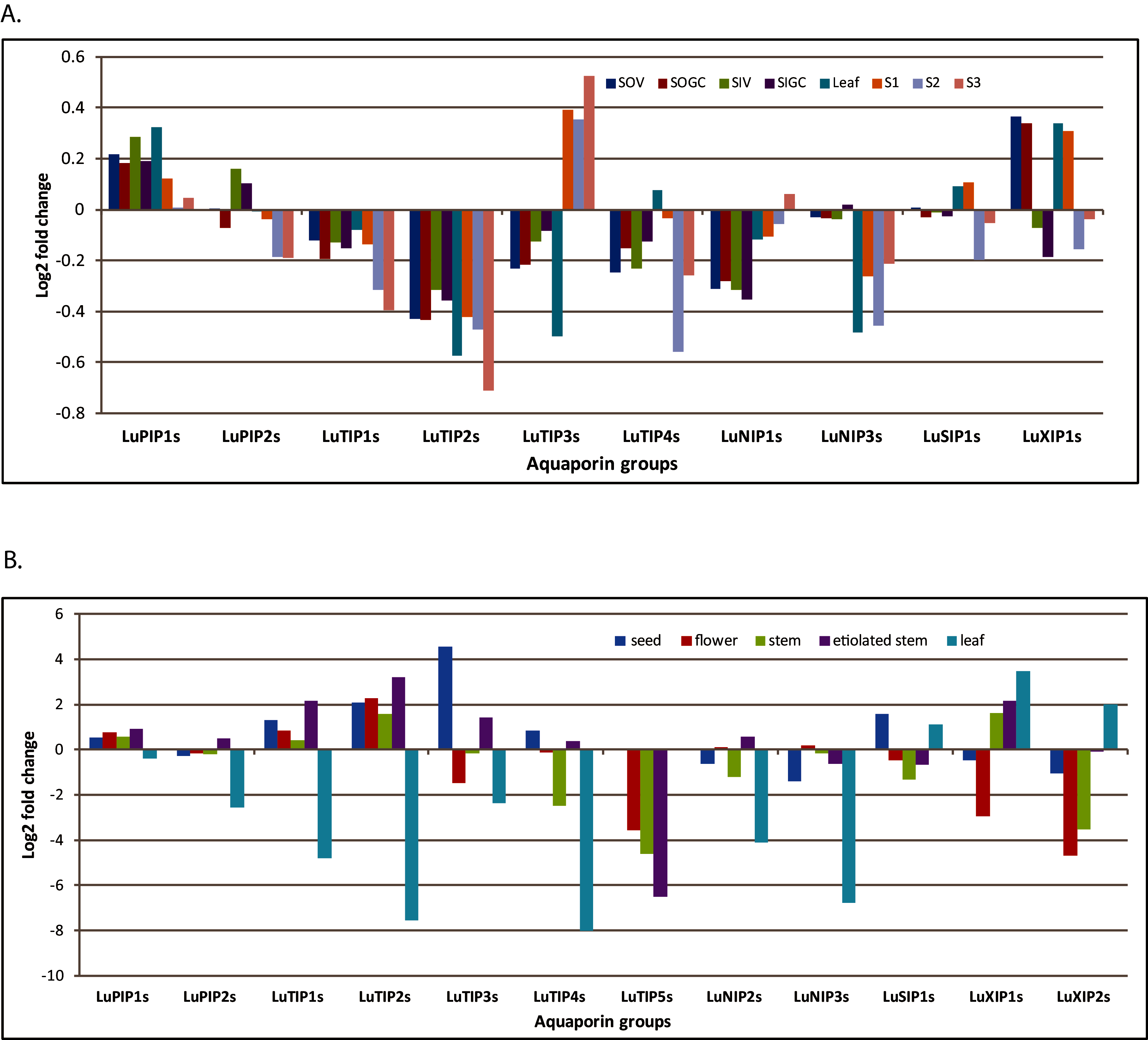
**

**Supplementary Figure S4.** Fold change of aquaporins (AQPs) in different tissues compared to root using microarray data (A) and RNA-Seq data (B). Analysis of flax AQPs expression using microarray data showing higher level of expression of TIP3 in seeds.

| 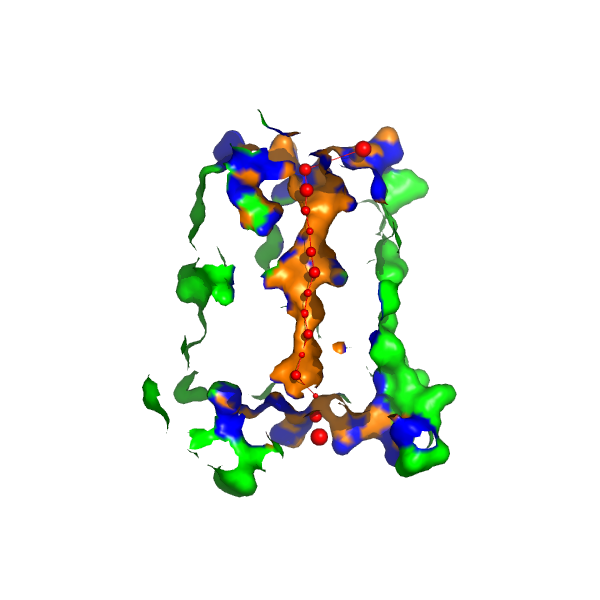 | 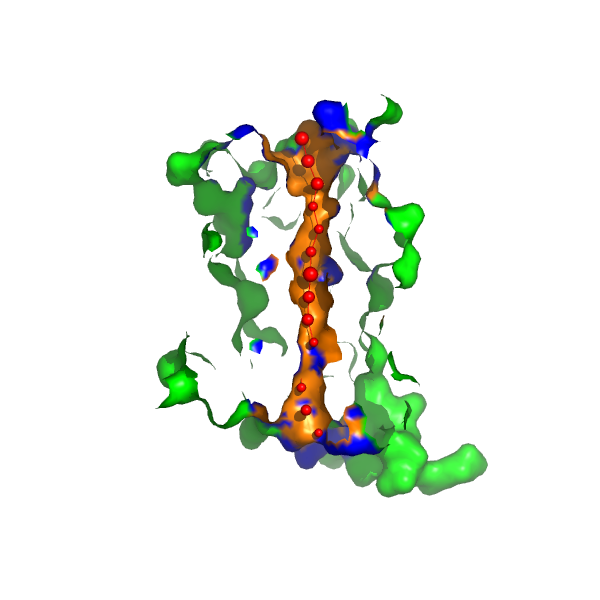 | 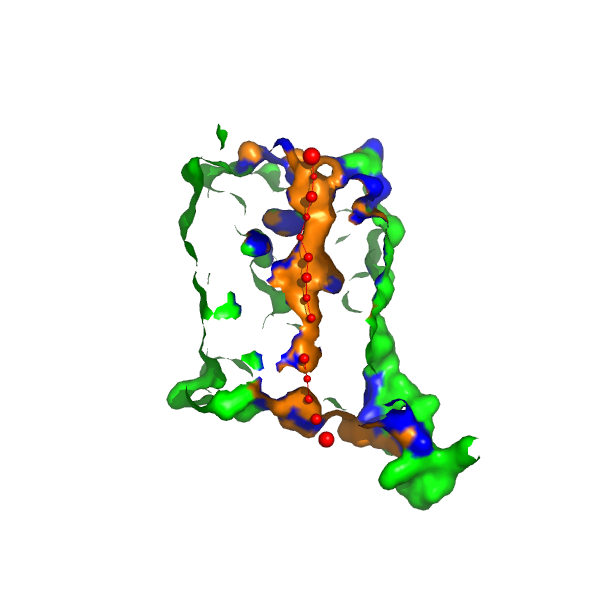 |
| --- | --- | --- |
| LuNIP1_1 | LuNIP1_2 | LuNIP1_3 |
| 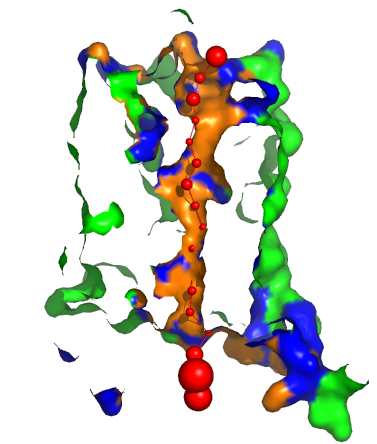 | 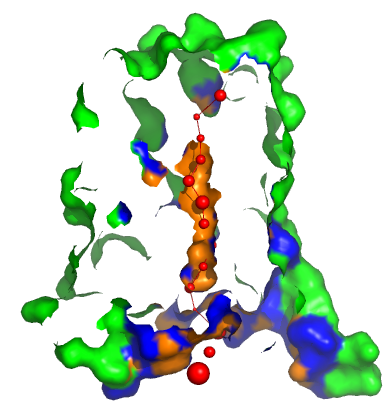 | 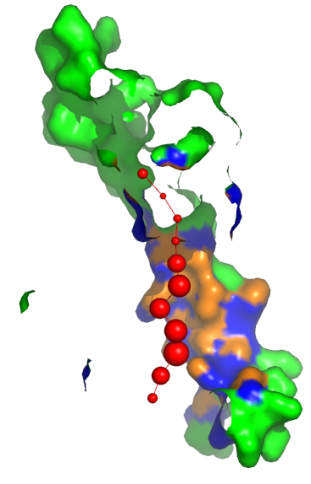 |
| LuNIP1_4 | LuNIP1_5 | LuNIP1_6 |
| 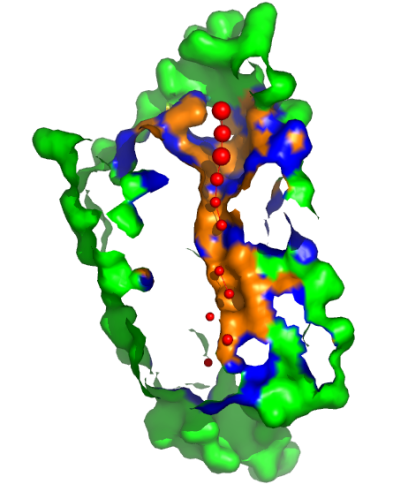 | 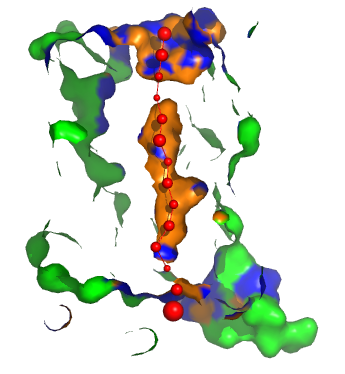 | 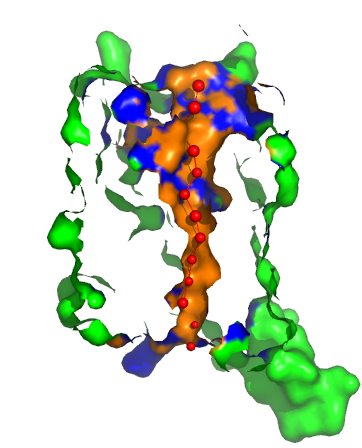 |
| LuNIP3_1 | LuNIP3_2 | LuNIP3_3 |

| 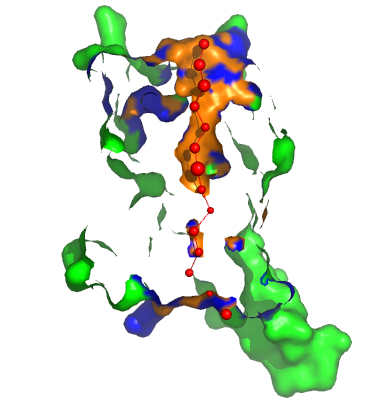 | 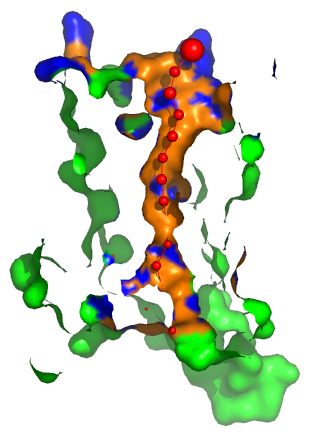 | 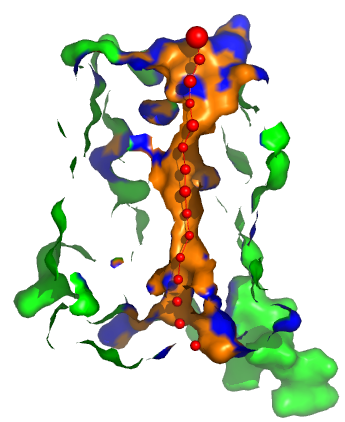 |
| --- | --- | --- |
| LuNIP3_4 | LuNIP3_5 | LuNIP3_6 |
| 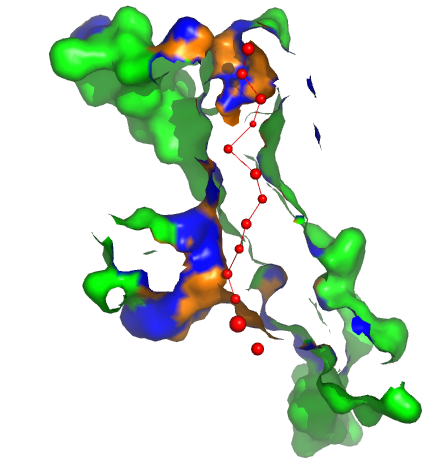 | 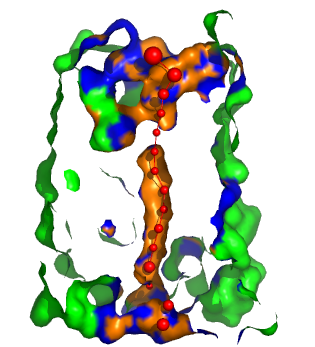 | 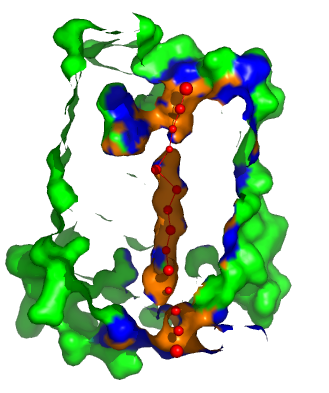 |
| LuNIP3_7 | LuPIP1_1 | LuPIP1_2 |
| 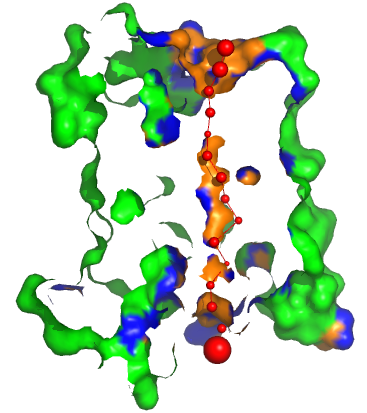 | 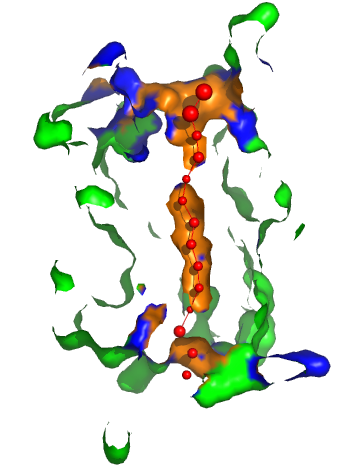 | 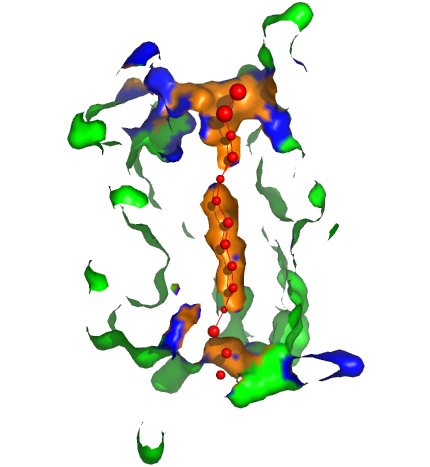 |
| LuPIP1_3 | LuPIP1_4 | LuPIP1_5 |

| 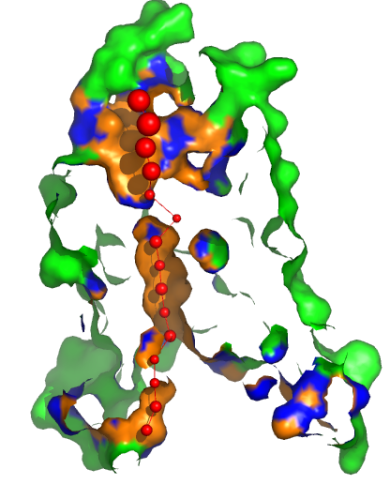 | 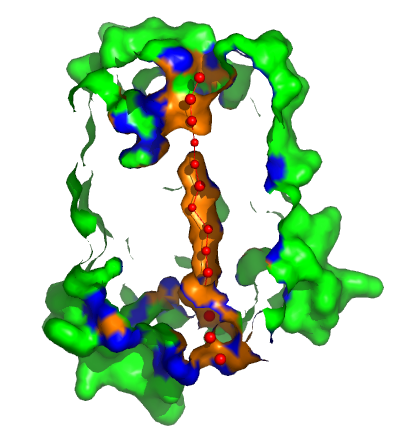 | 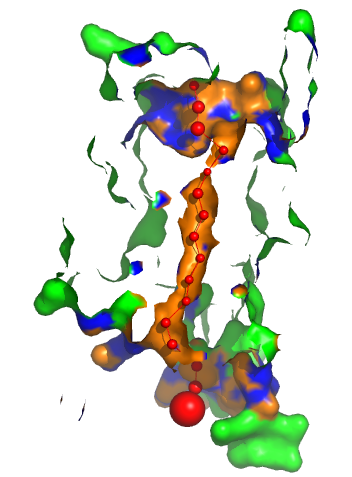 |
| --- | --- | --- |
| LuPIP2_1 | LuPIP2_2 | LuPIP2_3 |
| 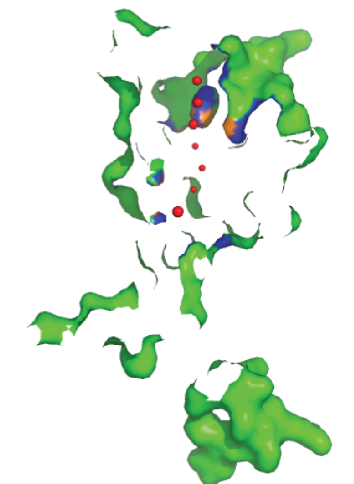 | 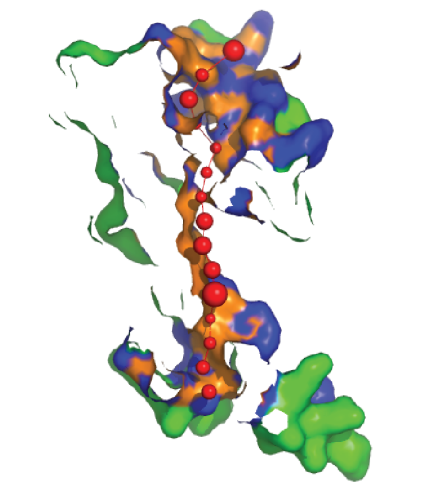 | 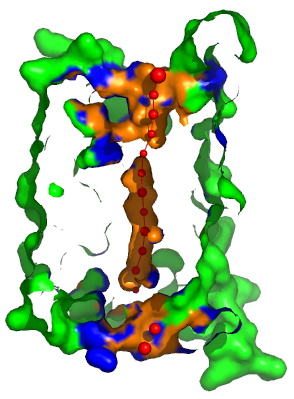 |
| LuPIP2_4 | LuPIP2_5 | LuPIP2_6 |
| 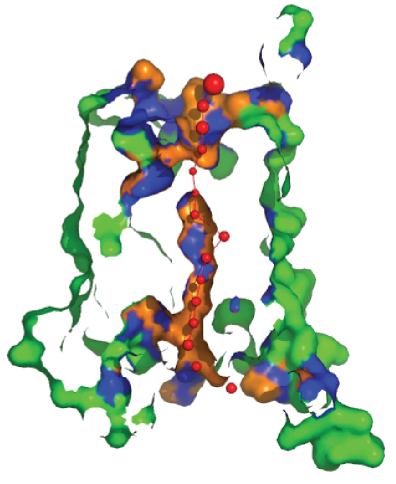 | 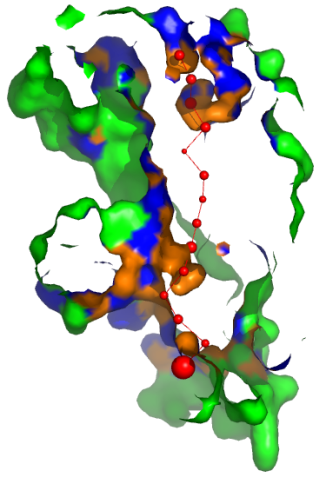 | 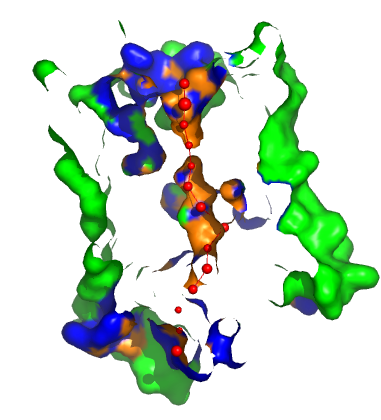 |
| LuPIP2_7 | LuPIP2_8 | LuPIP2_9 |

| 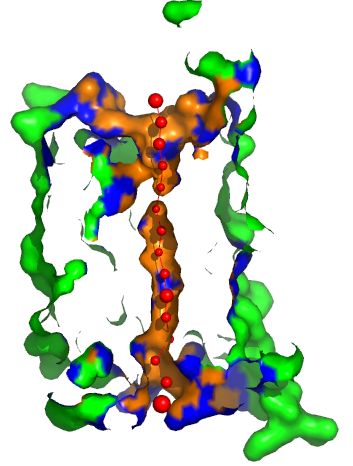 | 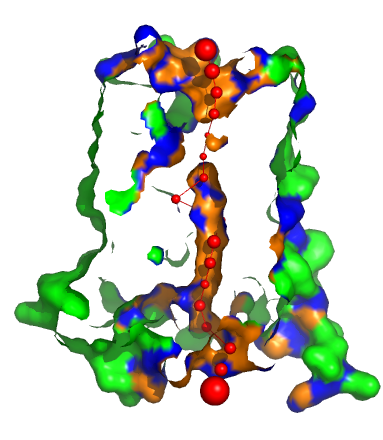 | 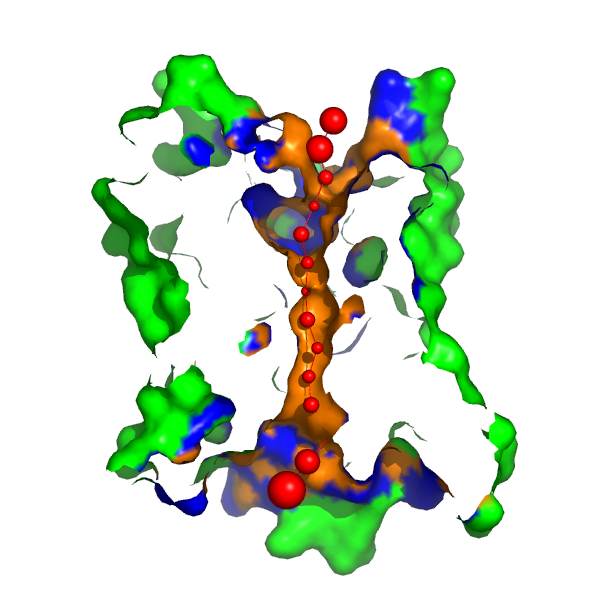 |
| --- | --- | --- |
| LuPIP2_10 | LuPIP2_11 | LuSIP1_1 |
| 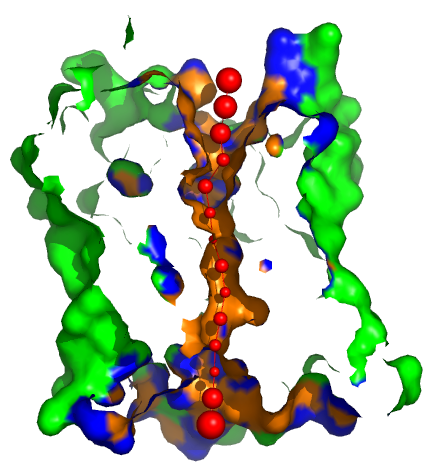 | 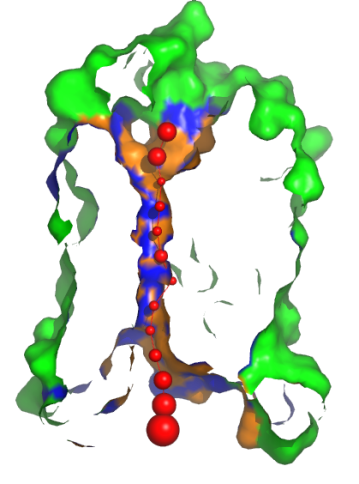 | 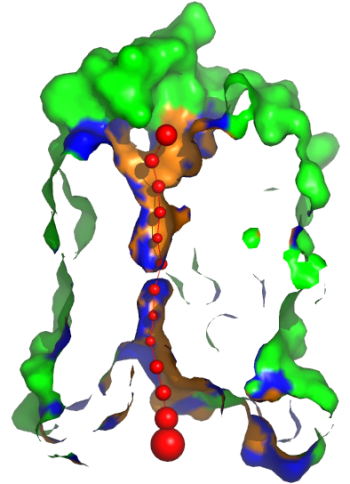 |
| LuSIP1_2 | LuTIP1_1 | LuTIP1_2 |
| 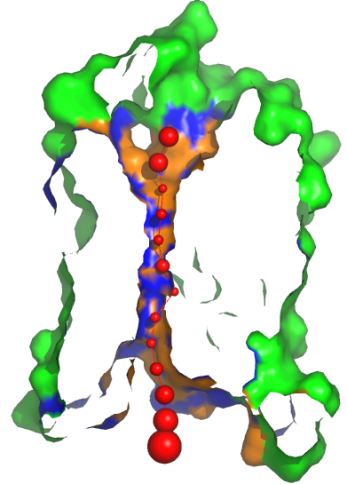 | 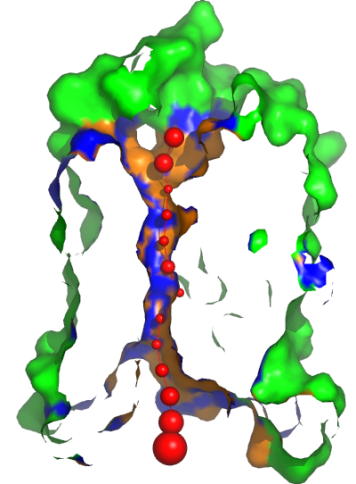 | 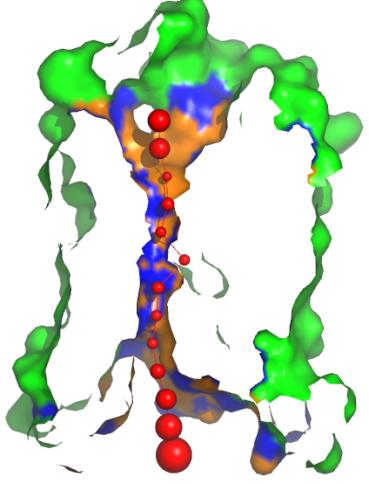 |
| LuTIP1_3 | LuTIP1_4 | LuTIP1_5 |

| 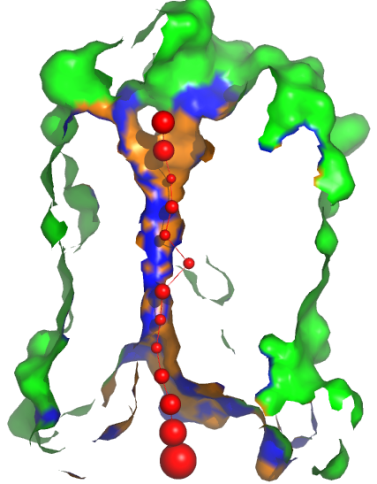 | 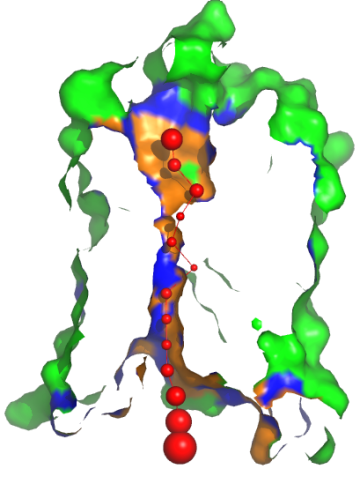 | 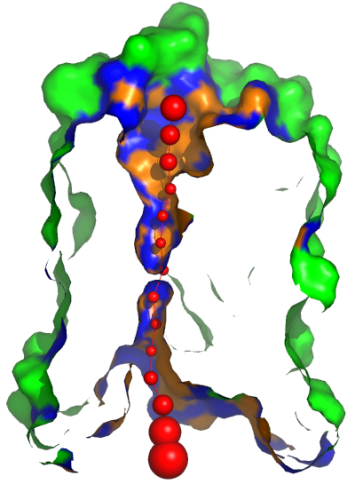 |
| --- | --- | --- |
| LuTIP1_6 | LuTIP1_7 | LuTIP2_1 |
| 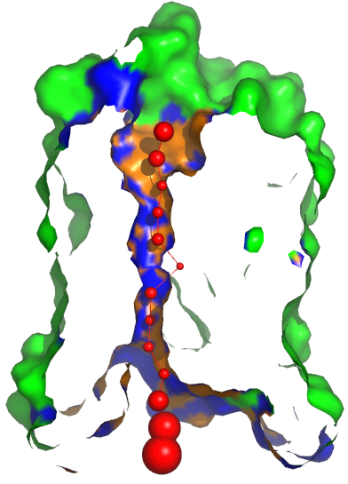 | 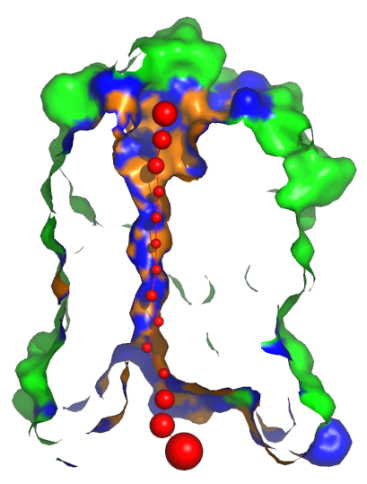 | 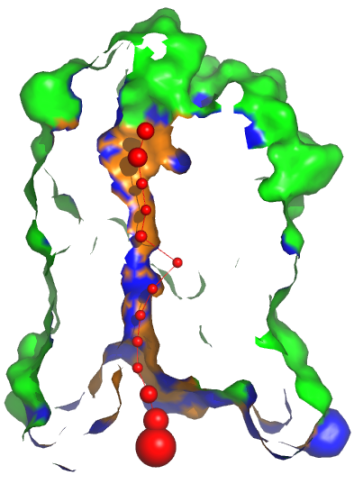 |
| LuTIP2_2 | LuTIP2_3 | LuTIP2_4 |
| 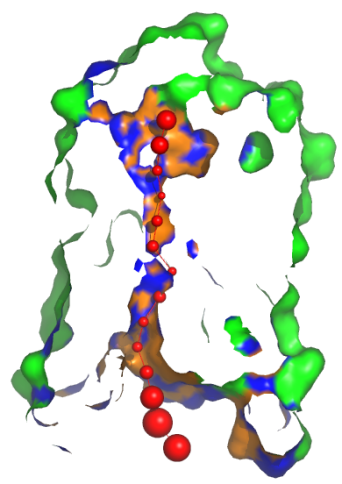 | 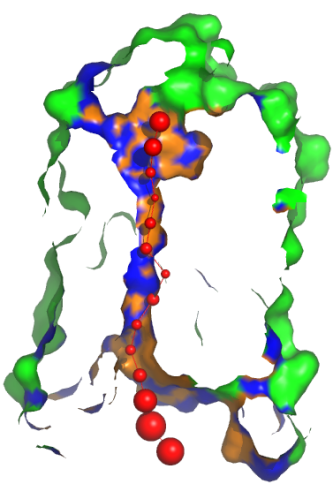 | 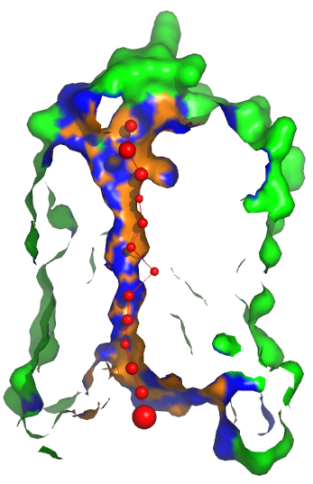 |
| LuTIP3-1 | LuTIP3-2 | LuTIP3-3 |

| 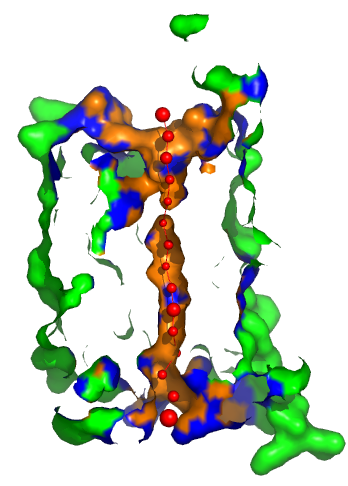 | 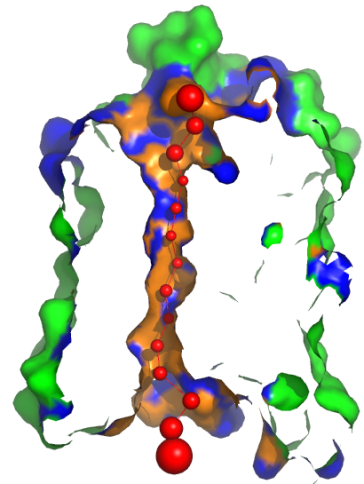 | 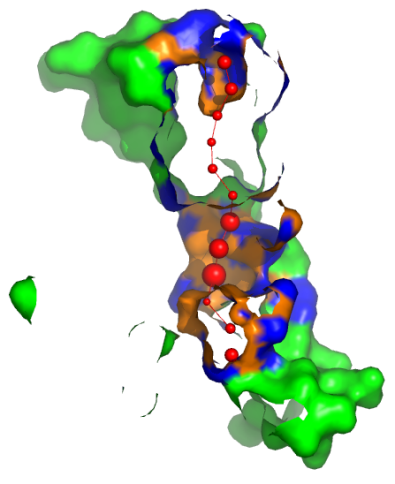 |
| --- | --- | --- |
| LuTIP3-4 | LuTIP4_1 | LuTIP5_1 |
| 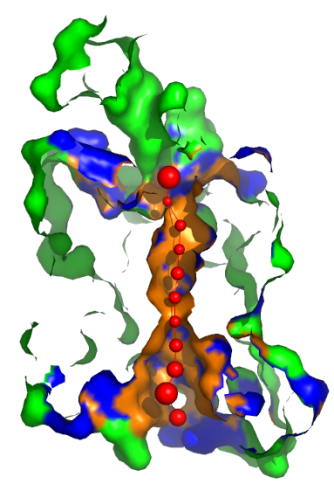 | 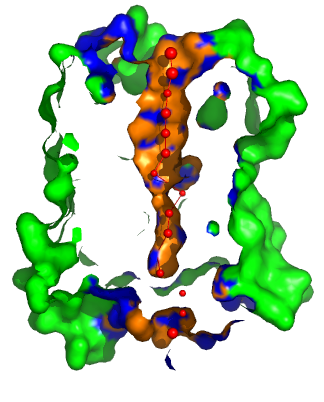 | 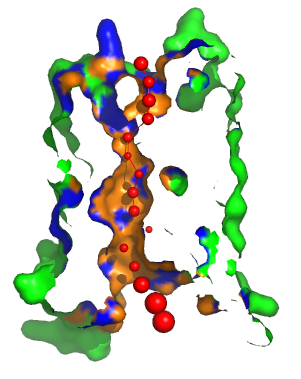 |
| LuXIP1_1 | LuXIP1_2 | LuXIP2_1 |

**Supplementary Figure S5.** Predicted structure of complete set of 51 aquaporins of flax classified into NIPs (13), PIPs (16), SIPs (02), TIPs (17), and XIPs (03).

**
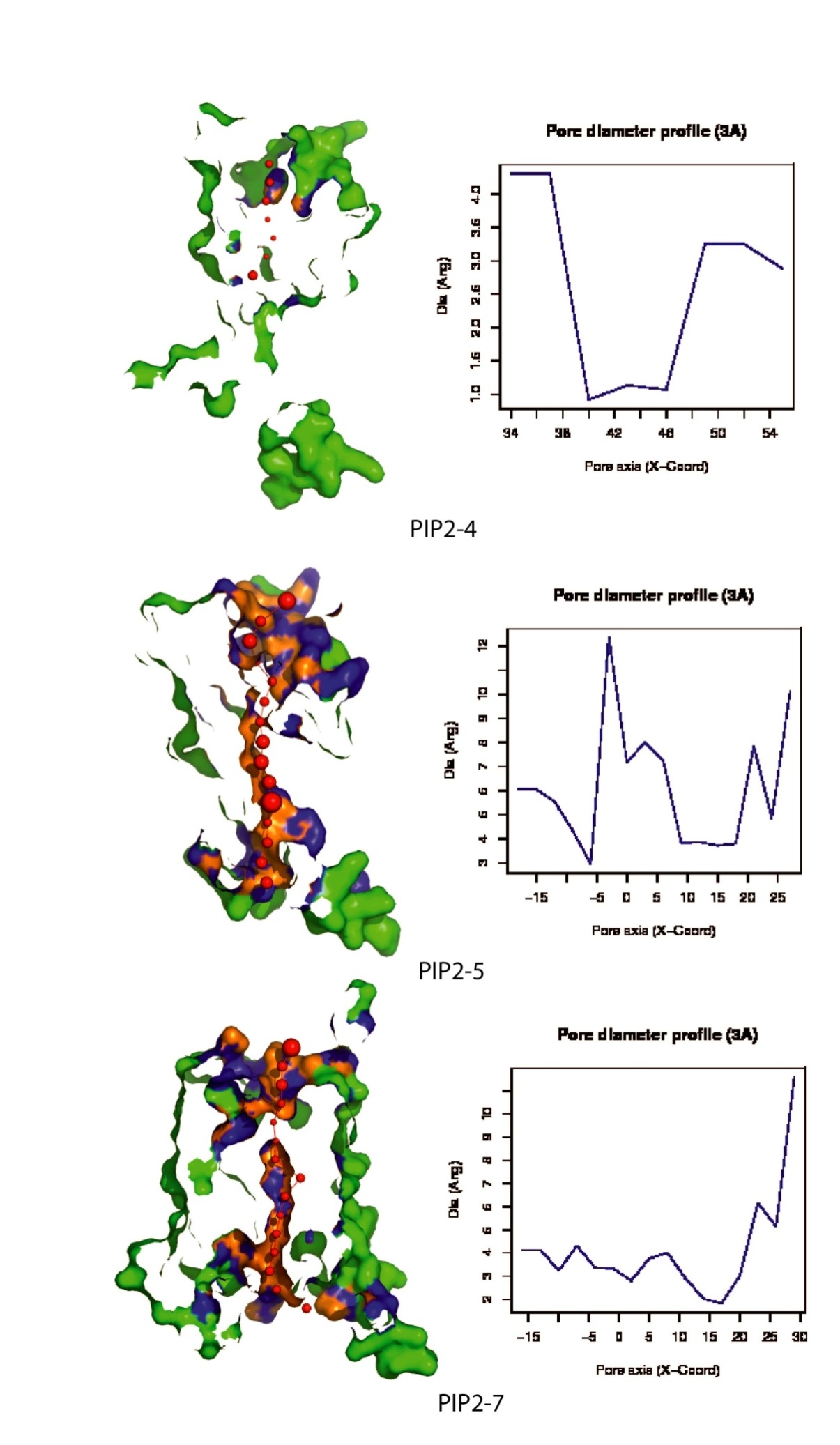
**

**Supplementary Figure S6.** Protein tertiary structure showing pore morphology of LuPIP2 family members. Cross section of the proteins showing pore was depicted for each family member along with the graph showing pore dimensions obtained from PoreWalker software.
